# Haplotype-resolved genomes of geminivirus-resistant and geminivirus-susceptible African cassava cultivars

**DOI:** 10.1186/s12915-019-0697-6

**Published:** 2019-09-18

**Authors:** Joel-E. Kuon, Weihong Qi, Pascal Schläpfer, Matthias Hirsch-Hoffmann, Philipp Rogalla von Bieberstein, Andrea Patrignani, Lucy Poveda, Stefan Grob, Miyako Keller, Rie Shimizu-Inatsugi, Ueli Grossniklaus, Hervé Vanderschuren, Wilhelm Gruissem

**Affiliations:** 1 Department of Biology, Institute of Molecular Plant Biology, ETH Zurich, Universitätstrasse 2, 8092 Zurich, Switzerland; 2 Functional Genomics Center Zurich, Winterthurerstrasse 190, 8057 Zurich, Switzerland; 3 Institute of Plant Biology, University of Zurich, Zollikerstrasse 107, 8008 Zurich, Switzerland; 4 Department of Evolutionary Biology and Environmental Studies, University of Zurich, Winterthurerstrasse 190, 8057 Zurich, Switzerland; 5 AgroBioChem Department, University of Liège, Passage des Déportés 2, Gembloux, Belgium; 6 Advanced Plant Biotechnology Center, National Chung Hsing University, 145 Xingda Road, Taichung, 40227 Taiwan

**Keywords:** Cassava genomes, Cassava mosaic disease, Haplotigs, Optical mapping, Chromosome proximity ligation, Transposable elements, Allelic expression

## Abstract

**Background:**

Cassava is an important food crop in tropical and sub-tropical regions worldwide. In Africa, cassava production is widely affected by cassava mosaic disease (CMD), which is caused by the African cassava mosaic geminivirus that is transmitted by whiteflies. Cassava breeders often use a single locus, *CMD2*, for introducing CMD resistance into susceptible cultivars. The *CMD2* locus has been genetically mapped to a 10-Mbp region, but its organization and genes as well as their functions are unknown.

**Results:**

We report haplotype-resolved de novo assemblies and annotations of the genomes for the African cassava cultivar TME (tropical *Manihot esculenta*), which is the origin of *CMD2*, and the CMD-susceptible cultivar 60444. The assemblies provide phased haplotype information for over 80% of the genomes. Haplotype comparison identified novel features previously hidden in collapsed and fragmented cassava genomes, including thousands of allelic variants, inter-haplotype diversity in coding regions, and patterns of diversification through allele-specific expression. Reconstruction of the *CMD2* locus revealed a highly complex region with nearly identical gene sets but limited microsynteny between the two cultivars.

**Conclusions:**

The genome maps of the *CMD2* locus in both 60444 and TME3, together with the newly annotated genes, will help the identification of the causal genetic basis of *CMD2* resistance to geminiviruses. Our de novo cassava genome assemblies will also facilitate genetic mapping approaches to narrow the large *CMD2* region to a few candidate genes for better informed strategies to develop robust geminivirus resistance in susceptible cassava cultivars.

**Electronic supplementary material:**

The online version of this article (10.1186/s12915-019-0697-6) contains supplementary material, which is available to authorized users.

## Background

As a subsistence crop, cassava is valued for its starchy storage roots, especially by small-holder farmers, because the plant produces starch even under unfavorable environmental conditions. Cassava is also becoming increasingly important as an industrial crop and as livestock feed [1, 2]. But genetic gains from breeding in cassava have made little progress over the last century compared to other crops [3]. The heterozygous genome, long breeding cycles, clonal propagation, and poor asynchronous male and female flowering have limited substantial genetic improvement [4].

In Africa and India, cassava mosaic disease (CMD) is the most important economic threat for cassava production. The whitefly-transmitted virus is spreading and affecting agricultural productivity as a result of substantial yield losses in CMD-susceptible cultivars, in extreme cases up to 100% [5, 6]. An estimated 25 million tons of cassava storage roots are lost to CMD annually, impacting food security for more than 500 million people [7–9].

To date, only four geminivirus resistance genes (R-genes) have been identified, mapped, cloned, and characterized in crops [10–13], indicating that only a small proportion of the natural genetic diversity for geminivirus disease resistance has been exploited. For cassava, only three known genetic resistance loci present in the germplasm are currently providing relatively stable field resistance to CMD. These are the polygenic, recessive *CMD1* locus that was introgressed from wild cassava relatives [14], the single-dominant gene locus *CMD2* in tropical *Manihot esculenta* (TME) cultivars that confers resistance to all known CMVs [15, 16], and the resistance source *CMD3* that was distinguished from *CMD2* recently based on a single marker [17].

Because a single-dominant gene greatly facilitates breeding, the *CMD2* locus became the predominant resistance source deployed in African cassava breeding programs, although its underlying molecular mechanism and robustness are currently unknown. *CMD2* was discovered in landraces collected from farmer fields in Nigeria and other West African countries during the 1980s and 1990s, but the breeding pedigrees of these landraces are unknown [15]. Recently, the breakdown of the *CMD2* resistance during tissue culture-induced embryogenesis, which is an essential step in cassava transformation, was reported for TME cultivars [18]. The fact that many geminivirus resistance breeding programs rely on the stability of the *CMD2* locus makes it urgent to understand its genome organization and function. This can be achieved using high-quality de novo genome sequences for African cassava cultivars to fully exploit the importance of this resistance source.

Efficient crop plant genome sequencing is often constrained by genome size and heterozygosity as well as the excessive proportion of repetitive DNA elements (RE). The cassava genome has a haploid genome size of approximately 750 Mb [19], but its heterozygosity is among the highest found in sequenced plant genomes [20] and it is rich in REs. Thus, cassava genomes have proven difficult to assemble and to date only highly fragmented and incomplete genome assemblies are available [19–21]. The first cassava draft genome from the partly inbred South American genotype AM560 [21] was released in 2012, followed by draft genomes of an Asian cassava cultivar KU50 and the cassava wild relative W14 (*Manihot esculenta* ssp. *flabellifolia*) [20]. These genetic resources enabled first population genomic studies [16, 22–24], transcriptome characterization [25–27], and whole methylome profiling [28]. However, the current versions of the draft cassava genomes are represented as linear, haploid DNA sequences. Such a representation for highly heterozygous genomes can cause misleading results when using read mapping-sensitive applications that rely on accurate read placement [29]. For example, whole-transcriptome sequencing reads can align falsely or even fail to map when they span challenging regions with structural variations (SVs). Misplaced reads do in turn result in both missed true variants or incorrectly reported false variants and bias subsequent results.

Here we report the long read-based de novo assembled genomes of CMD-susceptible and *CMD2*-resistant African cassava cultivars as diploid-nature, haplotype-resolved chromosome assemblies. They were generated using single-molecule, real-time sequencing (SMRT; Pacific BioSciences) to assemble long haplotypes that cover multiple heterozygous regions. The continuity of the long-read genome assemblies was subsequently improved by contig scaffolding using long-range linking information from optical maps (BioNano) [30] and chromosomal conformation capture (Hi-C) [31, 32]. Furthermore, we generated full-length mRNA sequencing (Iso-Seq) to correct and improve predicted gene models. The two African cassava genome assemblies will facilitate the development of new heterozygous, haplotype-phased cassava reference-ready genomes and serve as a resource for the identification of causal *CMD2* resistance genes.

## Results and discussion

### Cassava genome sequencing, assembly, and chromosome-scale scaffolding

**Fig. 1:**
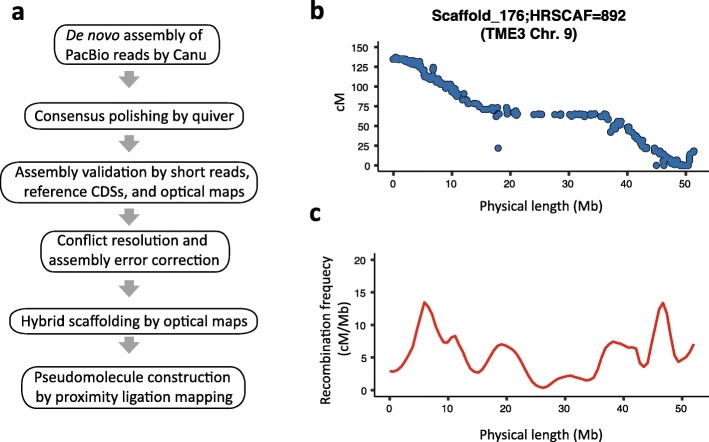
Assembly pipeline for the 60444 and TME3 African cassava genomes. **a** Overview of the processing pipeline used for the assemblies (see Additional file 3 for details). **b** Pseudomolecule validation using the location of SNP markers on the physical map (*x*-axis) as compared to their position on the composite cassava genetic map [33] (*y*-axis) for Chr.9, which is a single scaffold 176_TME3_. Each genetic marker is depicted as a dot on the plot (937 data points). **c** Graphical representation of mean local recombination frequencies between SNP markers along scaffold 176_TME3_. The *x*-axis represents the physical positions of the means on Chr 9, and the *y*-axis indicates the recombination ratio (centiMorgan (cM)/Mb) in each 1-Mb sliding window

We achieved a nearly complete de novo diploid assembly and annotation of the genomes for the African cassava cultivars 60444, which is CMD susceptible, and TME3 that carries the dominant *CMD2* resistance (Fig. 1). Using 70× PacBio whole genome shotgun long reads with N50 read length of 12,813 bp (60444) and 12,424 bp (TME3), we assembled the TME3 genome into 12,971 contigs with a N50 of 98 kb (i.e., 50% of the assembly consists of 98 kb or longer contigs). The 60444 genome was assembled into 11,459 contigs with a N50 of 117 kb (Table 1) (Additional file 1: Figure S1, Additional file 2: Table S1). We evaluated the performance of three different long-read assemblers (CANU-MHAP [34], FALCON v0.5 [35] and PBcR-MHAP [36]) by aligning Illumina paired-end (PE) reads to the corresponding long-read assemblies. This showed that the CANU assembler generated the most accurate assemblies, with the highest proportion of mapped paired-end (PE) reads (98.4% for 60444 and 96.4% for TME3) and the lowest proportion of discordant read-pair alignments (1.6% for TME3 and 1.2% for 60444) (Additional file 2: Table S2).

**Table 1:** Assembly statistics for the cassava TME3 and 60444 genomes compared with previously published assemblies of cassava genomes

Cultivar	TME3	60444	KU50 [20]	AM560 [19]
Number contigs	12,971	11,459	99,509	39,574
Contig N50 (kb)	97.58	116.8	5.28	27.87
Total contig length (Mb)	947	975	NA	NA
Total primary contig length (Mb)	732	713		
Total haplotig length (Mb)	213	260		
Optical map supported scaffolds	558	552	NA	NA
Primary scaffolds	506	491		
Optical Hybrid-scaffold N50 (Mb)	2.25	2.35	NA	NA
Hi-C scaffolding N50 (Mb)	53.35	59.19	NA	NA
Assembly size (Mb)	1225	1277	291.1*	582.3
TE proportion (%)	64.81	64.91	25.7	50.3
Annotated protein-coding genes	33,853	34,127	38,845	33,033

*The KU50 genome was reported to be 495 Mb in [20]; the number shown here was the published and downloadable DNA sequence available in 2014

The total length of assembled contigs was above 900 Mb for both TME3 and 60444. This was higher than the haploid genome size of approximately 750 Mb estimated by flow cytometry (Additional file 1: Figure S2), indicating that haplotypes of the heterozygous genomes were assembled independently into different contigs [37, 38]. Based on contig alignments against each other and read depth of coverage, we reassigned allelic contigs as primary contigs and haplotigs using Purge Haplotigs [39]. The total size of the de-duplicated primary haploid assembly was 732 Mb for TME3 and 713 Mb for 60444 (Table 1), which was close to the flow cytometry measurement (Additional file 1: Figure S2). The secondary haplotig assembly was more than 200 Mb. This reflects the high heterozygosity within the cassava genome, which is the consequence of interspecific admixture and past breeding, but short runs of homozygosity are also present in the genome [19, 40]. In this case, optical mapping is useful to phase haplotypes, especially in genomes with divergent homologous chromosomes [41]. We generated two high-coverage optical maps (150× for 60444, 130× for TME3) using the BioNano Genomics IrysView DNA imaging and analysis platform. The fluorescently labeled DNA molecules of the two cassava genomes assembled into similarly sized genomes of 1205 Mb for TME3 and 1204 Mb for 60444. This indicates that most of the parental chromosomes had been “phased” into haplotype segments by optical mapping (Additional file 2: Table S3). To further improve sequence contiguity and haplotype phasing, the PacBio contigs were corrected, joined, ordered, and oriented according to the optical mapping data. This generated a set of 558 optical-map-supported scaffolds spanning 634.1 Mb with a scaffold N50 of 2.25 Mb for TME3. For 60444, we generated 552 scaffolds spanning 714.7 Mb with an even higher scaffold N50 of 2.35 Mb.

The Portuguese introduced cassava from South-America to Africa in the sixteenth and seventeenth century, and since then the African germplasm diversity has remained exceptionally narrow [42]. Previous diversity studies relied on short-read mapping data only, but genome-wide structural variants are challenging to detect in heterozygous and complex plant genomes. The diploid optical maps from the two African cassava cultivars were tested for genomic diversity. The vast majority (81%) of the consensus optical maps from TME3 could be aligned with those from 60444 via common label patterns, indicating a very low level of structural diversity between the two cassava genomes. We then screened the alignments for TME3-specific insertions and deletions (INDELs) and identified evidence for 1058 insertions and 1021 deletions with average sizes of 57.4 kb and 45.7 kb, respectively (Additional file 2: Table S4).

### Genome completeness and haplotype phasing

**Fig. 2:**
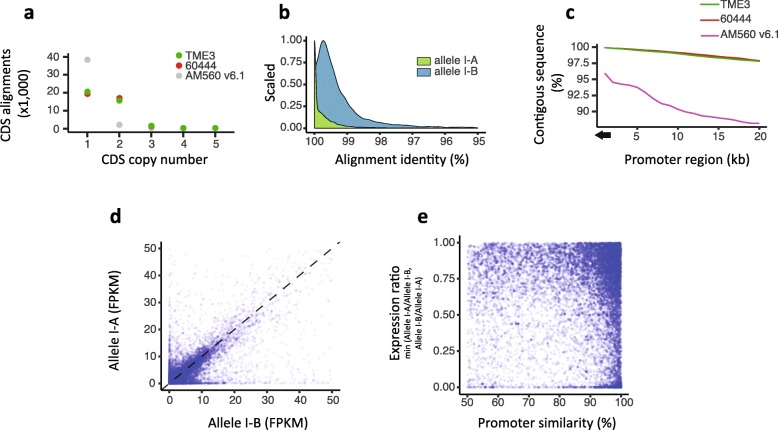
Haplotype phasing, allele nucleotide diversity, and allele-specific expression analysis for haplotype-aware cassava genomes. **a** Cassava CDS collection (*n* = 41,381) and their alignment copy number distribution in the two African cassava genomes TME3 (green points) and 60444 (red points), and the AM560 v6.1 genome (gray points). **b** Sequence alignment properties for the bi-allelic reference CDSs (*n* = 13,425) found in the 60444 genome. Bi-allelic genes, depicted as allele I-A and allele I-B, are presented as a green curve, and the homologous allelic counterpart as a blue curve. Percentage of alignment identity is shown on *x*-axis and data point density on the *y*-axis. **c** Promoter sequence contiguity (“N”-free-sequence) comparison between three different cassava genomes measured using 1-kb bins over a 20-kb region upstream the transcriptional start site. **d** Scatterplot of allele-specific gene expression in 60444 based on RNA read counts measured as fragments per kilobase of sequence per million mapped reads (FPKM). A bi-allelic gene is depicted as a single blue dot. Expression of one allelic copy is shown on the *x*-axis and the expression of the homologous counterpart on the *y*-axis. **e** Bi-allelic gene expression as a function of promoter sequence similarity. The bi-allelic gene expression ratio (*y*-axis) of 1.00 indicates an equal expression of both alleles, whereas the expression ratio of < 0.25 indicates mono-allelic expression (*n* = 3451). The promoter sequence similarity between the homologous alleles measured for a 2-kb region upstream of the start codon is shown on the *x*-axis. Bi-allelic genes with identical or near-identical promoter sequences can have mono-allelic expression

Haplotype phasing, or identifying alleles that belong to the same chromosome, is a fundamental problem in genetics. Our assembly strategy using PacBio long reads in combination with BioNano optical maps produced haplotype-aware genomic scaffolds in which phase information over long regions of homozygosity and even across assembly gaps was resolved. To further assess the completeness and quality of phased haplotypes in the two cassava genomes, publicly available cassava coding DNA sequences (CDSs) [19] were aligned to each of the assembled optical scaffolds using GMAP [43], which takes into account exon-intron junctions. Local duplicates, i.e., inter-scaffold matches, and CDSs with < 99% alignment coverage were removed from the analysis. Of the 41,381 CDS, 99.93% are present in the 60444 and TME3 genomes with only a few missing (84 and 86, respectively). This CDS alignment was used to estimate the haplotype phasing and allele number variation. In total, we detected 18,831 and 19,501 multi-copy gene loci in TME3 and 60444, respectively, with a large proportion of CDS aligning into allelic pairs (*n* = 15,679 for TME3 and *n* = 17,019 for 60444) (Fig. 2a).

Centuries of cassava clonal propagation has resulted in genetically fixed deleterious mutations that affect crop vigor and strongly limit breeding [3, 44, 45]. Duplicated regions are often subject to dynamic changes, including the accumulation of point mutations that facilitate species diversification [46]. To test this hypothesis for the bi-allelic genes in the diploid 60444 and TME3 genomes, we measured the nucleotide diversity for each allelic pair as determined by AM560 CDS alignments and plotted the proportion of single-base pair mutations. This analysis revealed high variation between coding sequences of alleles, further substantiating the heterozygosity within the coding portion of the genome (Fig. 2b) (sequence alignment mean: allele I-A 99.26%, allele I-B 97.15%).

Short-read-based genome assemblies frequently do not capture intergenic sequences that might be important for gene regulation because promoter regions often adjoin repetitive DNA sequences. Investigating gene expression regulation is highly dependent on accurately assembled promoters. We screened the promoter regions of bi-allelic genes and analyzed their sequence contiguity over a 20-kb region upstream the translational start codon (Fig. 2c). This revealed near complete promoter regions in the 60444 and TME3 genomes as compared to the AM560 v6.1 genome. The extensive sequence contiguity will facilitate allele-specific expression analysis and the identification of novel tissue-specific cassava promoter sequences.

To determine if the accumulation of allelic mutations has an impact on gene expression, we measured allele-specific expression using high-throughput RNA-seq analysis from eight sequencing libraries that originated from different tissues (for details, see Additional file 3). In total, we covered the expression of 18,723 genes with two alleles and identified 3451 (14.43%) genes with mono-allelic expression (Fig. 2d, e). Various mono-allelic expressed genes (44.76%) have highly similar promoter sequences (mean similarity = 95.52%) between the alleles, indicating that mono-allelic expression of these genes could be caused by one or more SNPs or may be epigenetically regulated through DNA methylation or chromatin packaging. It has been suggested that cassava developed a more robust maintenance methylation mechanism than found in other crop plant species [28]. The high number of alleles not expressed in the analyzed tissues could be another property of the cassava genome that was maintained through clonal propagation of the crop over centuries.

### Assembling pseudochromosomes of heterozygous cassava genomes

In cassava, a single bi-parental cross rarely yields enough progeny to generate a robust and dense genetic map that can be used to genetically anchor sequences to chromosomal pseudomolecules. The most recent publicly available cassava composite genetic map was generated from various mapping populations and anchors only 71.9% of an earlier haploid genome assembly [33]. To re-construct the set of cassava chromosomes independently of a composite genetic map (i.e., de novo), we generated chromosome proximity ligation libraries (Hi-C) for the TME3 and 60444 cassava cultivars (for details, see Additional file 3). Proximity mapping was previously shown to be instrumental for chromosome-scale assemblies in other species [31, 32]. The optical-map-improved scaffolds were combined with the remaining contigs and grouped according to the Hi-C-based molecule interaction maps using Dovetail proprietary algorithms. The approach has already been used recently in other crop genome sequencing projects to generate pseudochromosomes from the assembly of contigs and smaller scaffolds into contiguous scaffolds of chromosome size [47, 48]. Implementing the Dovetail assembly for cassava increased sequence contiguity by nearly 25-fold for a final scaffold N50 of 53.4 Mb in the TME3 and 59.2 Mb in the 60444 in African cassava genomes.

To assess the quality of the Hi-C-based chromosomal pseudomolecules, we aligned the genetic markers from the cassava composite genetic map [33]. Out of 22,403 genetic markers, we were able to align 22,341 (99.7%) with the 60444 genome and 22,373 (99.8%) with the TME3 genome. To visualize and validate the chromosomal pseudomolecules, we plotted the genetic distance against the physical distance for each genetic marker. At this level of resolution, these plots confirm that whole pseudochromosomes were assembled without large inter-chromosomal re-arrangements (Fig. 1b, Additional file 1: Figure S4). Plotting the recombination rate using a sliding window of 1 Mb across assembled scaffolds revealed the expected decrease in recombination frequency in the center of the scaffold, as well as the presence of other regions with low recombination in the chromosome arms (Fig. 1c, Additional file 1: Figure S5).

When analyzing the fasta sequences of the cassava pseudochromosomes in more detail, we found TME3 and 60444 pseudochromosomal scaffolds to contain more DNA sequence compared to the AM560 genome (Additional file 1: Figure S6). For example, Scaffold 7_TME3_ and Scaffold 1478_60444_ representing chromosome 12 were 107.1% and 116.3% larger than the chromosome 12 in AM560. The total length of the TME3 and 60444 pseudochromosomes was 29% greater than the haploid genome size estimated by flow cytometry, respectively. The additional sequences originate from repetitive sequences or spacers that were added by Dovetail in the assembly process but also represent coding sequences and gene models as well. When aligning the haploid composite genetic map [33] to the genome, we noticed that for loci where both haplotypes were assembled as allelic contigs/scaffolds, Hi-C scaffolding tended to integrate both haplotypes into pseudochromosomes, thus inflating genome size. We identified 78% of the genetic markers in TME3 (82.8% in 60444) as perfect hits (100% identity and coverage). Of those, 29.1% were present more than once in the TME3 genome (29.8% of 60444) (Additional file 1: Figure S7). Such a multiplication was expected, since both TME3 and 60444 are heterozygous genomes. We analyzed the various genome assemblies and found that the numbers of genetic markers that were present more than once were constant throughout the assembly process. In the CANU and CANU-BNG assemblies of both TME3 and 60444, the genetic markers are predominantly on different contigs and scaffolds, confirming that haplotypes have been assembled into separate allelic sequences. This is different in the Dovetail pseudochromosomes (Additional file 1: Figure S4), where 54.8% of TME3 and 56.5% of 60444 genetic markers can be found on contiguous sequences more than once (Additional file 1: Figure S7 E–F), indicating that both haplotpyes have been lifted up into Hi-C scaffolds. Co-location of genetic markers on the same scaffold was not a local phenomenon but was spread over the entire genome. For example, on scaffold 7_TME3_ representing presudochromosome 12 (Additional file 1: Figure S8), 2635 genetic markers are aligned twice or more, while they were mostly separated on allelic sequences in the CANU-BNG assemblies, indicating integration of both haplotypes in the Dovetail pseudochromosome (Additional file 4: Table S5). Copies of the same genetic marker typically occur in close proximity to each other, with a median distance of 343 kb. A remaining set of 87 genetic markers was already duplicated on individual contigs of scaffold 7_TME3_ in the initial CANU assembly of chromosome 12 and thus likely represent true gene duplication events. They were on average separated by 27.9 kb with up to eight gene copies per contig in some cases. After removing the duplicated allelic sequences in the Dovetail pseudochromosomes based on haplotig purging (Additional file 2: Tables S6 and S7), the total size of the pseudochrosomes was 796 Mb for TME3 and 854 Mb for 60444.

Proximity ligation mapping was also used to identify miss-joints and mis-assemblies. Based on the Hi-C data, we identified 30 mis-assemblies in the TME3 optical map scaffolds and only 16 in the 60444 scaffolds. Each mis-assembly was validated manually by testing Hi-C read-pair alignment positions and alignment depth, and scaffolds were corrected accordingly (Additional file 1: Figure S9). However, the proximity maps of TME3 and 60444 will be valuable for quality assessment of the composite genetic map and to improve the sequence resolution in regions that are seemingly devoid of meiotic recombination.

### Repetitive DNA analysis and genome annotation of cassava pseudochromosomes

**Fig. 3:**
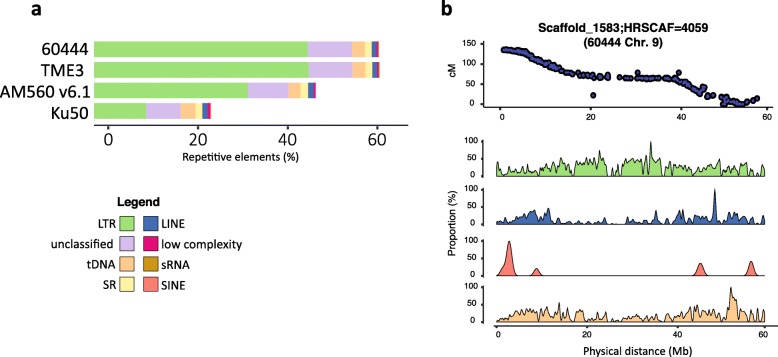
Distribution of major repetitive elements identified in cassava genomes. **a** Percentage of base pairs of assembled cassava genomes that represent long terminal repeat (LTR), unclassified repeat, DNA transposon (tDNA), sources of short RNA (sRNA), long interspersed elements (LINE), low-complexity element, and short interspersed nuclear element (SINE) sequences. **b** Graphical representation of SNP markers (top) and chromosomal density plots for the four predominant TE categories (bottom) on the scaffold 1583_60444_ map

Transposable elements (TEs) and REs are involved in genome evolution and shaping gene regulatory networks [49]. Unlike previous sequencing technologies, SMRT reads can span and resolve entire TE and RE regions [50]. Using de novo generated cassava DNA repeat libraries, we annotated up to 2.5 times more TEs (defined by REPEATMASKER and REPEATMODELER, as described in the “Methods” section) in the pseudochromosomes compared to earlier reports [19–21] (Fig. 3a). In the TME3 and 60444 Dovetail assemblies, we annotated 602.90 Mb (64.81%) and 633.93 Mb (64.91%) as repetitive sequences, respectively. As an example, we investigated the spatial distribution of sequence repeats along the entire chromosomal scaffold 1583_60444_, which corresponds to pseudochromosome 9 (Fig. 3b) and generated density maps for the four predominant TE categories. Long terminal repeat (LTR) retrotransposons have higher densities in the centromer region, while non-LTR retrotransposons elements (LINE and SINE) are clustered in telomere-proximal regions. Class II DNA transposons are more equally distributed across that scaffold. A similar distribution of TEs was reported for other complex plant chromosomes [51, 52], confirming the high quality of cassava genome sequences ordered using Hi-C. Our pseudochromosome assemblies reveal a high proportion of repetitive DNA in cassava (65% of total contig length), which is similar to the amount of repetitive DNA found in other sequenced complex crop genomes such as sorghum (54%) [53], quinoa (64%) [54], or barley (81%) [52] (detailed TE annotation in Additional file 2: Table S9).

We predicted protein coding and microRNA (Additional file 2: Table S10) sequences using a combination of ab initio prediction and transcript evidence from available cassava gene models [19]. Protein-coding sequence annotation was assisted by Iso-Seq (high-quality, full-length cDNAs from single-molecule sequencing) data that covered 15,478 (45.7%) gene loci in TME3 and 16,057 (47.0%) in 60444 (Additional file 1: Figure S10). The quality of the gene model annotation was assessed for 1440 conserved plant genes using BUSCO [55]. We found 95% of the single-copy conserved orthologs in both genomes, with only 20 and 19 genes partially assembled in TME3 and 60444, respectively (Additional file 2: Table S11).

### Protein expansion in cassava genomes

**Fig. 4:**
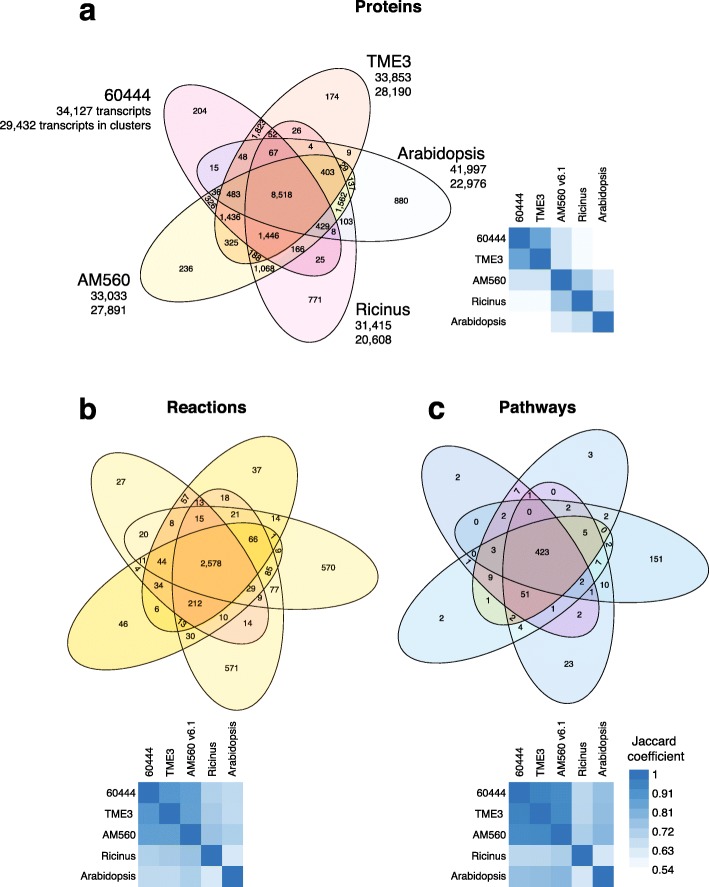
Expansion of gene clusters, enzymatic reactions, and metabolic pathways. **a** Associations of protein groups using OrthoMCL clustering, **b** predicted metabolic reactions, and **c** metabolic pathways present in the cassava 60444, TME3, and AM560 genomes and in the genome of their close relative *Ricinus communis* as well as the *Arabidopsis thaliana* genome as outgroup. Numbers in the Venn diagram sections correspond to the number of cluster groups. The first number below the cultivar name denotes the total number of transcripts for proteins that were included in the OrthoMCL analysis. The second number indicates the number of transcripts from the genes for the proteins contained in the protein clusters. The heatmaps show the Jaccard coefficient between two species (intersection divided by the union of their proteins, reactions, or pathways)

The two African cassava cultivars 60444 and TME3 are thought to have exceptional low genetic diversity [19]. The similar number of annotated genes allowed us to investigate gene family expansions specific to the two cultivars. We used OrthoMCL clustering of all gene models present in our two assemblies as well as the genome assemblies of the South American cassava cultivar AM560, *Ricinus communis* as a close relative of cassava, and *Arabidopsis thaliana* as an outgroup [56, 57]. This confirmed that the two African cassava cultivars are closely related (Fig. 4a). For example, there were fewer gene family groups specific to 60444 or TME3 (0.8–1.1%), whereas the number of specific gene family groups was considerably larger for *Ricinus* and *Arabidopsis*. Interestingly, there were more protein groups associated exclusively with AM560 and *Ricinus* than with *Ricinus* and either 60444 or TME3. These trends were also seen for predicted enzymatic reactions (Fig. 4b) and predicted metabolic pathways (Fig. 4c) but, as expected, overall the four species were similar for total reactions and metabolic pathways [57].

There remained 1823 protein groups containing 4081 gene models (2067 for 60444 and 2014 for TME3) that are specific to the two African cassava genomes. Considering the short evolutionary time since cassava was introduced to Africa about 400 years ago, it is likely that the differences in gene divergence and expansions between AM560, 60444, and TME3 evolved before the ancestor or ancestors of 60444 and TME3 was brought to the African continent.

We subsequently investigated genes of proteins associated with gene families for overrepresentation of GO terms [58]. For AM560, we found cultivar-specific proteins with GO terms enriched for “polygalacturonase activity” (Additional file 1: Figure S11). Among the most significantly enriched GO terms for genes that were associated exclusively with the African cultivars were categories “structural integrity of ribosomes” (GO:0003735) and “structural molecule activity” (GO:0005198) (Additional file 1: Figure S12). Another more specific function was squalene monooxygenase activity (GO:0004506). Interestingly, single-strand DNA virus infection increases squalene production [59]. Squalene monooxygenase converts squalene to (3S)-2,3-epoxy-2.3-dihydrosqualene (epoxysqualene), which is a precursor for many specialized metabolites (Additional file 1: Figure S13). Both in 60444 and TME3, there are four metabolic pathways predicted to be involved in the conversion of epoxysqualene to several specialized metabolites. Some have known antimicrobial, anti-inflammatory, and/or anti-tumor activities, including beta-amyrin that can be converted to oleanolate, which has antiviral activity [60] and inhibits topoisomerase I/II [61], which are involved in replication of viruses such as cauliflower mosaic virus (CaMV) [62] . The Rep locus in the CMD-related mungbean yellow mosaic virus (MYMV) encodes a protein with topoisomerase activity [63]. Since the Rep locus is found in all Gemini viruses, functionality is likely conserved [64]. The pathway from squalene to oleanolic acid involves three consecutive reactions that all have gene annotations in all three cassava cultivars. The two African cultivars 60444 and TME3 that are exposed to CMGs, however, have an expanded gene pool for two of the three reactions in the pathway (Additional file 1: Figure S12).

### *CMD2* locus

**Fig. 5:**
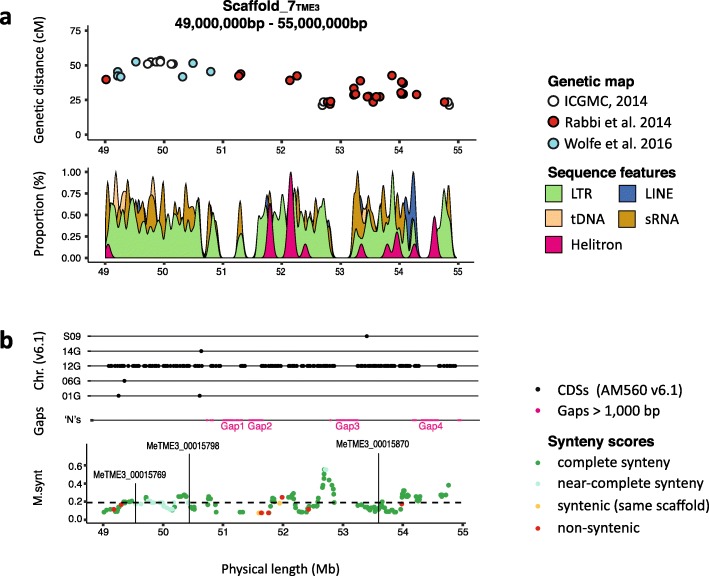
*CMD2* locus in TME3 genome. **a** The upper panel shows *CMD2*-associated genetic SNP markers and their genetic distance relative to their physical position on scaffold_7 of TME3. Red dots indicate *CMD2* SNP markers released by Rabbi and colleagues [16], and blue dots indicate the SNP markers released by Wolfe and colleagues [22, 42]. The lower panel shows the distribution of main repetitive genomic features at the *CMD2* locus. **b** The upper panel shows the alignment position of AM560 v6.1 CDS in the region of Chr. 12 containing the *CMD2* locus. Each black dot represents the CDS alignment position at the *CMD2* scaffold (*x*-axis) and its chromosomal origin from the AM560 v6.1 cassava reference genome. Sequence breaks (gaps > 1 Kb) are shown as pink bars. The lower panel shows the MSS for every annotated gene at the *CMD2* locus in TME3. Green dots indicate genes that are found in the *CMD2* region of 60444, and light blue dots indicate genes that are found in close proximity of the *CMD2* locus in 60444. Orange dots indicate TME3 genes that show a syntenic relation to 60444 genes on other 60444 scaffolds, and red dots indicate genes with no syntenic relation. The dashed line represents the MSS average for the whole genome

The identification and molecular characterization of geminivirus resistance genes in cassava has been slowed by missing genomic resources. Previous genetic mapping placed the *CMD2* locus in separate regions of AM560-2 (v6.1) chromosome 12 [16, 22], suggesting that precise *CMD2* mapping is difficult because of few recombination events and borderline marker saturation. We found that genetic markers released from these mapping efforts aligned to an approximate 5-Mb region between 49 and 55 Mb of scaffold 7_TME3_ (Fig. 5a). The same markers were identified on 60444 scaffold 1478_60444_.

Analysis of the *CMD2* locus in scaffold 7_TME3_ revealed that nearly all markers from a bi-parental mapping population [16] aligned to a region between 51 and 55 Mb (Fig. 5a, red circles, with a single marker outside of this region at 49 Mb) and the marker set that had been generated from an association mapping approach [22] spanned an adjacent region of approximately 3 Mb (49–51 Mb) in the same scaffold (Fig. 5a, blue circles). These results suggest that the genetic marker sets that previously identified two separate loci in fact correspond to a single region spanning 6 Mb of scaffold 7_TME3_. However, the pseudochromosome 12 region containing the *CMD2* locus has four major assembly gaps (Fig. 5b), which likely result from extensive stretches of repetitive DNA that prevent complete assembly of the region. The alignment of the AM560 CDS in the *CMD2* region revealed high conformity with the AM560 chromosome 12 to scaffold 7 of TME3 containing the *CMD2* locus (Fig. 5b). In 60444, the markers aligned with a 6-Mb region on Scaffold 1478_60444_.

To better understand the similarity between the 60444 and TME3 genomes, we analyzed their synteny and in particular synteny in the region of the *CMD2* locus using the Comparative Genomics platform (CoGe) (Additional file 1: Figure S14). More than 70% of the genes encoded within the *CMD2*_TME3_ locus were found to be syntenic to a gene within the *CMD2*_60444_ and *CMD2*_AM560_ loci (Fig. 5b, Additional file 1: Figure S15). Less than 10% of the genes either had no syntenic gene (red) in the other two genomes or the syntenic genes were outside the *CMD2* locus in a larger region three times the size of the *CMD2* locus. Two TME3 genes, MeTME3_00015756 and MeTME3_00015762, are missing from the *CMD2* regions of AM560 and 60444, both short gene models of unknown functions. While at the level of microsynteny most genes are syntenic, the organization of the *CMD2* locus is not entirely contiguous between the TME3, 60444, and AM560 genomes except for a region with high microsynteny around 52.7 Mb. It is unlikely that the low organizational microsynteny is the result of pseudochromosome mis-assemblies because genes between 52.1 and 54.7 Mb of *CMD2*_TME3_ are found on a single CANU-BNG scaffold with low microsynteny to the corresponding regions in AM560 and in 60444.

We searched our de novo gene annotations in the *CMD2* loci of the TME3 and 60444 chromosome 12 scaffolds for three suggested CMD resistance candidate genes that were identified in the AM560 v6.1 genome [22]. Manes.12G076200 and Manes.12G076300 encode peroxidases, a protein class that is involved in many biochemical reactions [65]. In tomato, peroxidase activity increases in juvenile leaves during whitefly-mediated geminivirus infections [66]. We confirmed the presence of the two peroxidase genes (MeTME3_00015769 and MeTME3_00015798) at the *CMD2* locus of 60444 and TME3. Manes.12G068300 encodes a protein disulfide-isomerase-like 2-3 (PDI). This type of enzyme catalyzes the correct folding of proteins and prevents the aggregation of unfolded or partially folded precursors. We identified MeTME3_00015870 in the *CMD2* locus of TME3 that encodes a similar PDI. In barley, genetic studies identified HvPDI5-1, which is the ortholog of MeTME3_00015870, as a virus susceptibility factor that contributes to resistance to bymoviruses [67].

When expanding the search proximal and distal to the *CMD2* locus for genes that could provide resistance to geminivirus infection, we identified a gene encoding Suppressor of Gene Silencing 3 (SGS3, MeTME3_00015743, 1.71 Mb downstream of the *CMD2* locus). SGS3 is involved in posttranscriptional gene silencing (PTGS) and functions together with RNA-directed RNA polymerase 6 (RDR6) during dsRNA synthesis [68]. SGS3 has also been suggested to function in the transport of the RNA-silencing signal [69]. SISGS3, the tomato homolog of Arabidopsis SGS3, interacts with the tomato yellow leaf curl geminivirus (TYLCV) V2 protein that functions as a suppressor of silencing and counteracts the innate immune response of the host plant [70]. The identified genes provide useful information for candidate proteins related to the function of the dominant *CMD2* locus in protection against geminivirus infection in TME3 and other *CMD2*-type cassava cultivars.

## Conclusions

The diploid-aware de novo assemblies of the heterozygous 60444 and TME3 cassava genomes will help to unlock the limited genomic diversity of African cassava cultivars for crop improvement and geminivirus resistance breeding. The genome assembly strategy reported here can be similarly adapted to other medium-sized, non-inbred genomes with high heterozygosity and DNA repeat-rich regions. Using the information for haplotype-phased alleles and allele-specific expression, it will be possible to characterize and purge deleterious mutations using targeted genome editing [71], conventional breeding, or genomic selection. Moreover, the large haplotype scaffolds of the 60444 and TME3 genomes will greatly facilitate trait mapping and map-based cloning of agriculturally important genes in this important food security crop.

Our results show that the new maps of the *CMD2* locus in both 60444 and TME3, together with the newly annotated genes, will help to identify the causal genetic basis of *CMD2* resistance to geminiviruses. Our de novo genome assemblies will also facilitate genetic mapping efforts to narrow the large *CMD2* region to a few candidate genes for better informed strategies to develop robust geminivirus resistance in susceptible cultivars. Furthermore, the genome assemblies will lead to a better understanding of the genetic differences between cassava cultivars and how genetic variability can be deployed in breeding programs for future cassava improvement.

## Methods

Further details of all methods are presented in Additional file 3. No statistical methods were used to predetermine sample size. Experiments were not randomized, and investigators were not blinded to allocation during experiments and outcome assessment.

### Long-read sequencing and sequence assembly

To sequence the two cassava genomes with long reads, we extracted high molecular weight (HMW) genomic DNA from 3-week-old leaf tissue of in vitro grown cassava 60444 and TME3 plants following a modified protocol [72]. Libraries for PacBio SMRT sequencing were generated as described previously [73]. Libraries were sequenced using a PacBio RSII instrument with P6C4 sequencing reagents. We used 47 SMRT cells for TME3 and 45 SMRT cells for 60444. For 60444, we generated a total of 52.4 GB with subread bases with a mean read length of 12.8 kb. For TME3, 53.9 GB of subread bases were generated with a similar mean read length of 12.4 kb. The PacBio sequences had a > 70-fold genome coverage.

De novo assembly of the subreads was performed applying three assemblers: the PBcR-MHAP pipeline [36], the CANU-MHAP assembler [34], and the FALCON (v0.5) assemblers [35]. For FALCON, we adopted parameter sweeping and the assembly with the largest N50 was retained. For the other assemblers, default parameters were used, except the expected haploid genome size was set to values estimated by flow cytometry as well as k-mer analysis (Additional file 3). Quiver from SMRT Analysis v2.3.0 was run two times to polish base calling of assembled contigs [74].

### Optical map construction

Long-range scaffolding of the assembly contigs with optical mapping was achieved using the Irys optical mapping platform (BioNano Genomics). HMW DNA was isolated from 3-week-old leaf tissue of in vitro grown 60444 and TME3 cassava plants, embedded in thin agarose plugs according to the IrysPrep Kit and the plant tissue DNA isolation protocol (BioNano Genomics). DNA molecules were labeled using the *NT.BspQI* DNA-nicking enzyme by incorporation of fluorescent-dUTP nucleotides according to the IrysPrep nick-and-repair protocol (BioNano Genomics). DNA samples were aliquoted and quantitated using the Qubit Fluorimeter run in broad-range mode. The final samples were then loaded onto the IrysChips, linearized and visualized by the BioNano Irys molecule imaging instrument. Molecules > 150 kb were assembled de novo using the pairwise assembler provided by the IrysView software package (BioNano Genomics) with *p* value threshold of 10^−9^.

### Three-dimensional genome-wide chromatin capture sequencing

Freshly harvested leaves of in vitro grown cassava 60444 and TME3 plants were vacuum infiltrated in nuclei isolation buffer (NIB) supplemented with 2% formaldehyde. Protein crosslinking was stopped by adding glycine and applying an additional vacuum infiltration step. Leaf tissue was snap-frozen using liquid nitrogen and ground into a fine powder, re-suspend in NIB, and purified by spin-downs as described earlier [75]. Nuclei were digested with 400 units of *HindIII* as described in [75]. Digested chromatin was labeled using a fill-in reaction with 60 units of Klenow polymerase and biotin-14-dCTP. The exonuclease activity of T4 DNA polymerase was used to remove biotin-14-dCTP from non-ligated DNA ends. Proteinase K was added to reverse the formaldehyde crosslinking, and DNA was purified following phenol-chloroform extraction [75]. The Hi-C samples were quality assessed by PCR amplification of a 3C template and evaluated according to [75] (Additional file 1: Figure S3). Quality control passed Hi-C samples were purified following a phenol-chloroform extraction protocol [75] and mechanically sheared to fragment sizes of 300 bp using a Covaris S2 sonicator. Hi-C library fragments were blunt-ended using the End Repair Mix from Illumina and finally purified using AMPure beads according to the standard AMPure protocol. The biotinylated Hi-C samples were enriched through biotin-streptavidin-mediated pull-down and adenylated using Illumina’s A-tailing mix. Illumina paired-end sequencing adaptors were ligated to the Hi-C fragments, and a PCR amplification of the Hi-C library was carried on as suggested earlier [75]. Finally, PCR products were purified using AMPure beads following the standard AMPure protocol and quantified using a Qubit device. Samples were sequenced using the Illumina HiSeq 2500 instrument. This produced 385 million pairs of 150-bp reads for 60444 and 391 million reads for TME3 (Additional file 2: Tables S13 and S14). Genome scaffolding was performed with Dovetail Genomics’ HiRise scaffolding software.

### Assembly accuracy estimation, repeat identification, and gene annotation

Publicly available WGS Illumina paired-end reads [76] were trimmed and quality filtered using Trimmomatic [77] and mapped to the draft assembly using BWA ALN (v0.7.12) [78] with default parameters. WGS read-mapping files were sorted using SAMtools SORT [79] statistics and called using QUALIMAP BAMQC [80]. Identification allelic sequences in all drafts was performed using Purge Haplotigs [39] (Additional file 1: Figure S16). To assess the assembly completeness, the set of reference CDSs (https://phytozome.jgi.doe.gov/pz/portal.html#!info?alias=Org_Mesculenta) was aligned to each of the assembled draft genome using GMAP [43] with option “-no fails” and “min-identity 0.5.” Results were further filtered for alignments covering > 99% of query sequence using a custom script.

Repeat families found in the draft genome assemblies of 60444 and TME3 were first independently discovered de novo and structure classified using the software package REPEATMODELER ver. 1.0.9 and REPEATMASKER ver. 4.0.7 (https://www.repeatmasker.org). To screen for large tandem repeats, we used the software package RefAligner from Bionano with the option “-simpleRepeat -simpeRepeatTolerance 0.1 -simpleRepeatMinEle 3.”

To annotate the gene space, we performed iterative MAKER analysis. In the initiated analysis, the gene prediction tool AUGUSTUS [81] was trained with reference gene models. The predicted gene models were combined with alignment base evidence, including all ESTs from cassava found on NCBI (https://www.ncbi.nlm.nih.gov/nucest/?term=cassava%20ESTs), Iso-Seq data, and UniProt protein sequences. The initiated set of MAKER gene models were used to train gene predictor SNAP, which was added in the second round of MAKER analysis, together with gene predictor GeneMark trained using Iso-Seq data. Putative gene functions of the final set of gene models were characterized by performing a BLAST search of the protein sequences against the Uniprot database (ftp://ftp.ebi.ac.uk/pub/databases/fastafiles/uniprot/). PFAM domains, InterProScanID, and Gene Ontology annotation were obtained by running interproscan [82]. To annotate non-protein-coding genes, the tools tRNAscan-SE [83] and Infernal [84] were used together with the Rfam version 13.0 database.

### Allele-specific expression analysis and promoter region comparison

Newly generated RNA-seq datasets were derived from three key developmental stages of cassava 60444: early stage plant with fibrous root (FR) and leaf, middle stage plant with leaf, FR and intermediate root (IR), and late stage plant with leaf, FR, IR. RNA-seq libraries were sequenced using Illumina HiSeq2000 in paired-end 2 × 100 nucleotides mode. We aligned the RNA-seq reads using STAR [85] and retained the unique alignments. Reads were counted using SAMtools and custom made scripts [79].

Promoter regions were characterized for genes with two alleles and fpkm expression ratio > 0. Sequences 2 kb upstream of the start codon were defined as promoter. A pairwise alignment was generated for each allele pair using the MUSCLE pairwise alignment tool [86]. Alignments were analyzed using 100-bp bins, and a similarity ratio was calculated using a custom script and visualized using the INCHLIB cluster and heatmap tools [87].

### Genome-wide comparison and structural variation detection

To compare the 60444 and TME3 assemblies on a genome-wide scale, we used the optical maps of the two cassava cultivars to detect structural variations (SVs) using the RunBNG software [88]. We used the maps from 60444 as the reference and TME3 as query. RunBNG acts as a wrapper and essentially uses the BioNanos’ RefAligner for generating the alignments. Alignments were then screened using the script “SVdetect” to detect the intergenomic SVs and to calculate insertion and deletion sizes [73]. Synteny was analyzed using the CoGe platform (https://genomevolution.org/). Syntenic regions between 60444 and TME3 were identified using CoGe SynMap and SynFind. The resulting table contains all genes in TME3 and the syntenic genes that were detected in 60444. We then defined a microsynteny score for every gene j in TME3. In a window of m genes surrounding gene j, stretching maximally n genes upstream and maximally n genes downstream on the same scaffold, we calculated for every m gene the longest syntenic gene sequence where all genes are conserved syntenic in the same or antisense direction in 60444. For *n* = 5, the maximal value per gene is thus 11 if gene j has both 5 genes up and 5 genes downstream and all 11 genes can be found in the same or antisense order in 60444. We then summed all scores of the genes in the window and divided by the square of the number of genes. Thus, in a window of 11 genes ABCDEFGHIJKTME3 where ABCDETME3 can be found in 60444 on Scaffold 1 and FGHIJKTME3 on Scaffold 2, the score is 5 × 5 + 6 × 6/11^2^ = 0.504. The same scoring results of a gene duplication in one genome but not the other.

The QTL *CMD2* on 60444 and TME3 has been identified using BLAST alignments of markers from the composite genetic map of cassava [33] and screened for markers from scaffold5214 and scaffold06906. Scaffold5214 has been reported by Rabbi and colleagues [16] to be closely linked to *CMD2*, and Scaffold6906 has been revealed in an association study [22]. Best BLAST hits were filtered and plotted using custom R-scripts. To identify the *CMD2* region of the AM560 genome, we used BLAST searches using a subset of the genetic markers: (1) Rabbi et al. [16] marker S5214_780931, (2) Wolfe et al. [22, 42] (only those with a *p* value < 10–50) S8_5645072, S8_5801843, S8_5801851, S8_6106055, S8_6218789, S8_6222418, S8_7325190, S8_7325312, S8_7325397, S8_7717243, S8_7717285, S8_7762525, S8_7762556, S8_7790078, S8_7790133. The markers represent SNPs; thus, a 81-bp region (40 bp either side of the disease resistance associated SNP) was used for each BLAST search. For each SNP marker, we performed a manual investigation and a single hit was identified on chromosome 12 and the *CMD2* locus was defined 100,000 bp either side of these BLAST hits.

### Gene family analysis

To investigate gene family expansion specific in the 60444 or TME3 genomes, we used OrthoMCL clustering of all gene models present in our assemblies, the assembly of AM 560, the assembly of *Ricinus communis* as a close relative of cassava, and *Arabidopsis* as an outgroup [56, 57]. Only the longest protein sequence was selected, and datasets were filtered for internal stop codons. Pairwise sequence similarities between all input protein sequences were calculated using BLASTP [89] with an *e* value cutoff of 10^−5^. Clustering of the resulting matrix was used to define the orthology cluster with an inflation value set to 1.5. Over- and underrepresentation of Gene Ontology (GO) terms between the three cassava genomic compartments were calculated with a hypergeometric test using the functions GOstats and GSEABase from the Bioconductor R package [90]. The REVIGO [91] package was used to remove redundant and similar terms from long Gene Ontology lists by semantic clustering and to visualize the enrichment results. To define local duplicated genes, OrthoMCL clustering was used. Local duplicated genes were reported when one orthologous neighboring gene was encoded on the same scaffold with a maximum distance of 100 kb and a 10 gene interval.

Enzyme prediction and pathway prediction was performed as published earlier [57].

## Additional files

Additional file 1: **Figure S1.** Summary of data generated for genome construction. **Figure S2.** Genome size estimation for the two cassava genotypes using flow cell cytometry. **Figure S3.** Quality controls for the Hi-C libraries constructions. **Figure S4.** Pseudo-molecule validation using the 22,403 genetic markers from the cassava composite genetic map and the 18 pseudo-chromosomes of the cassava composite genetic map. **Figure S5.** Recombination rates for the cassava chromosomal pseudo-molecules. **Figure S6.** Plot of the length of the 18 chromosomes of AM560 compared to the combined length of the sequences that can be associated with the respective chromosomes in 60444 and TME3. **Figure S7.** Occurrence of genetic markers identified on TME3 and 60444. **Figure S8.** Genetic distance to physical distance plot of TME3 Scaffold 7, representing Chromosome 12 in AM560. **Figure S9.** Example of a mis-assembly identification using chromosome conformation capture read pairs. **Figure S10.** Summary of full-length transcriptome sequencing for high-quality gene-space annotation. **Figure S11.** GO enrichment analysis for the genes specific to the AM560 genome. **Figure S12.** GO enrichment analysis for the genes specific to the 60444 and TME3 genome. **Figure S13.** Squalene monooxygenase activity pathway and the corresponding gene models found in 60444, TME3 and AM560. **Figure S14.** Syntenic dotplot. **Figure S15.** Syntenic relation of the long arm of chromosome 12 between the AM560 v6.1 genome and equivalent scaffolds of the TME3 or 60444 genomes. **Figure S16.** Read coverage histograms of TME3 and 60444 assemblies. (DOC 11613 kb)

Additional file 2: **Table S1.** Assembly statistics of representative genome drafts from the three different assemblers. **Table S2.** Assembly accuracy evaluation using publicly available Illumina paired-end reads. **Table S3.** Optical map assembly using the IrysView software provided by BioNano and using option ‘optArguments_human’. **Table S4.** Structural variations based on optical maps of two cassava lines. **Table S8.** PacBio Iso-seq full length-transcriptome sequence classification. **Table S9.** Structural annotation of transposable elements in 60444 and TME3. **Table S10.** Non-coding RNA detected in the two cassava genomes. **Table S11.** BUSCO analysis of genome assemblies for 60444 and TME3. **Table S12.** Scaffolds representing the 18 pseudochromosomes of the cassava de novo genomes. **Table S13.** Hi-C library sequencing and read quality. **Table S14.** Hi-C read pair evaluation using HiCUP analysis pipeline (v0.5.8). (DOC 189 kb)

Additional file 3: Supplementary Materials and Methods. (DOC 177 kb)

Additional file 4: **Table S5.** Duplicated genetic markers in TME3 genome assemblies. **Table S6.** Duplicated allelic sequences (haplotigs) lifted up into TME3 pseudochromosome scaffolds by Dovetail using Hi-C data. **Table S7.** Duplicated allelic sequences (haplotigs) lifted up into 60444 pseudochromosome scaffolds by Dovetail using Hi-C data. (XLS 1479 kb)

## Data Availability

The cassava TME 3 and 60444 PacBio raw reads have been deposited at NCBI Short Read Archive (SRA) under BioProject number PRJEB27129 [92]. Genome assemblies and optical maps have been deposited at NCBI under BioProject number PRJNA508471 [93]. All other data are available from the corresponding authors upon reasonable requests. Public Illumina data sets SRX1393211 [94] and SRX526747 [76] were downloaded from NCBI SRA.

## Abbreviations

CaMV: Cauliflower mosaic virus
CDS: Coding DNA sequence
CM: Centimorgan
CMD: Cassava mosaic disease
FGCZ: Functional Genomic Center Zurich
FPKM: Fragments per kilobase of sequence per million mapped reads
FR: Fibrous root
GO: Gene Ontology
HMW: High molecular weight
INDELs: Insertions and deletions
IR: Intermediate root
LINE: Long interspersed element
LTR: Long terminal repeat
MYMV: Mungbean yellow mosaic virus
NCBI: The National Center for Biotechnology Information
NIB: Nucleus isolation buffer
PDI: Protein disulfide-isomerase
PE: Paired-end
PGDB: Plant genome database Japan
PTGS: Posttranscriptional gene silencing
RDR6: RNA-directed RNA polymerase 6
RE: Repetitive DNA element
R-genes: Resistance genes
SGS3: Suppressor of Gene Silencing 3
SINE: Short interspersed element
SMRT: Single-molecule, real-time sequencing
SRA: Short Read Archive
sRNA: Short RNA
SV: Structural variation
tDNA: DNA transposon
TEs: Transposable elements
TME: Tropical *Manihot esculenta*
TYLCV: Tomato yellow leaf curl geminivirus
