# Supplementary material for: Haplotype-resolved genomes of geminivirus-resistant and geminivirus-susceptible African cassava cultivars
Source: BMC Biol. 2019 Sep 18;17:75. doi: 10.1186/s12915-019-0697-6 (PMC6749633; doi:10.1186/s12915-019-0697-6)
Supplement: Supplementary file 1 — Figure S1. Summary of data generated for genome construction. Figure S2. Genome size estimation for the two cassava genotypes using flow cell cytometry. Figure S3. Quality controls for the Hi-C libraries constructions. Figure S4. Pseudo-molecule validation using the 22,403 genetic markers from the cassava composite genetic map and the 18 pseudo-chromosomes of the cassava composite genetic map. Figure S5. Recombination rates for the cassava chromosomal pseudo-molecules. Figure S6. Plot of the length of the 18 chromosomes of AM560 compared to the combined length of the sequences that can be associated with the respective chromosomes in 60444 and TME3. Figure S7. Occurrence of genetic markers identified on TME3 and 60444. Figure S8. Genetic distance to physical distance plot of TME3 Scaffold 7, representing Chromosome 12 in AM560. Figure S9. Example of a mis-assembly identification using chromosome conformation capture read pairs. Figure S10. Summary of full-length transcriptome sequencing for high-quality gene-space annotation. Figure S11. GO enrichment analysis for the genes specific to the AM560 genome. Figure S12. GO enrichment analysis for the genes specific to the 60444 and TME3 genome. Figure S13. Squalene monooxygenase activity pathway and the corresponding gene models found in 60444, TME3 and AM560. Figure S14. Syntenic dotplot. Figure S15. Syntenic relation of the long arm of chromosome 12 between the AM560 v6.1 genome and equivalent scaffolds of the TME3 or 60444 genomes. Figure S16. Read coverage histograms of TME3 and 60444 assemblies. (DOC 11613 kb) [file 12915_2019_697_MOESM1_ESM.doc]

**Supplementary Figures**

**Figure S1**

Summary of data generated for genome construction. a) Size distribution of PacBio SMRT RS II subreads from single-molecule sequencing of DNA from TME3 and 60444. b) Distribution of molecule lengths from BioNano Irys runs for TME3 and 60444. c) Sequence binning using the proximity data. The x- and y-axes give the mapping positions of the first and second read in the read pair. The color of each square gives the number of read pairs within that bin. White vertical and black horizontal lines have been added to show the borders between scaffolds. Scaffolds less than 1 Mb are excluded.

**Figure S2**

Genome size estimation for the two cassava genotypes using flow cell cytometry and the tomato haploid genome reference ‘Stupice’

**Figure S3**

Quality controls for the Hi-C libraries constructions from 60444 and TME3. Control for labeling and ligation of ends in Hi-C libraries. The ligation junction of two close genomic cassava HindIII fragments was PCR-amplified and digested. In the no fill-in controls, no NheI restriction site can be generated and the HindIII recognition site remained intact. In contrast, the Hi-C junctions derived from blunt-end ligation of filled-in HindIII sites were cleaved by NheI. DNA was separated using a standard 1.5% agarose gel. Sizes of the PCR-products are indicated on the left.

**
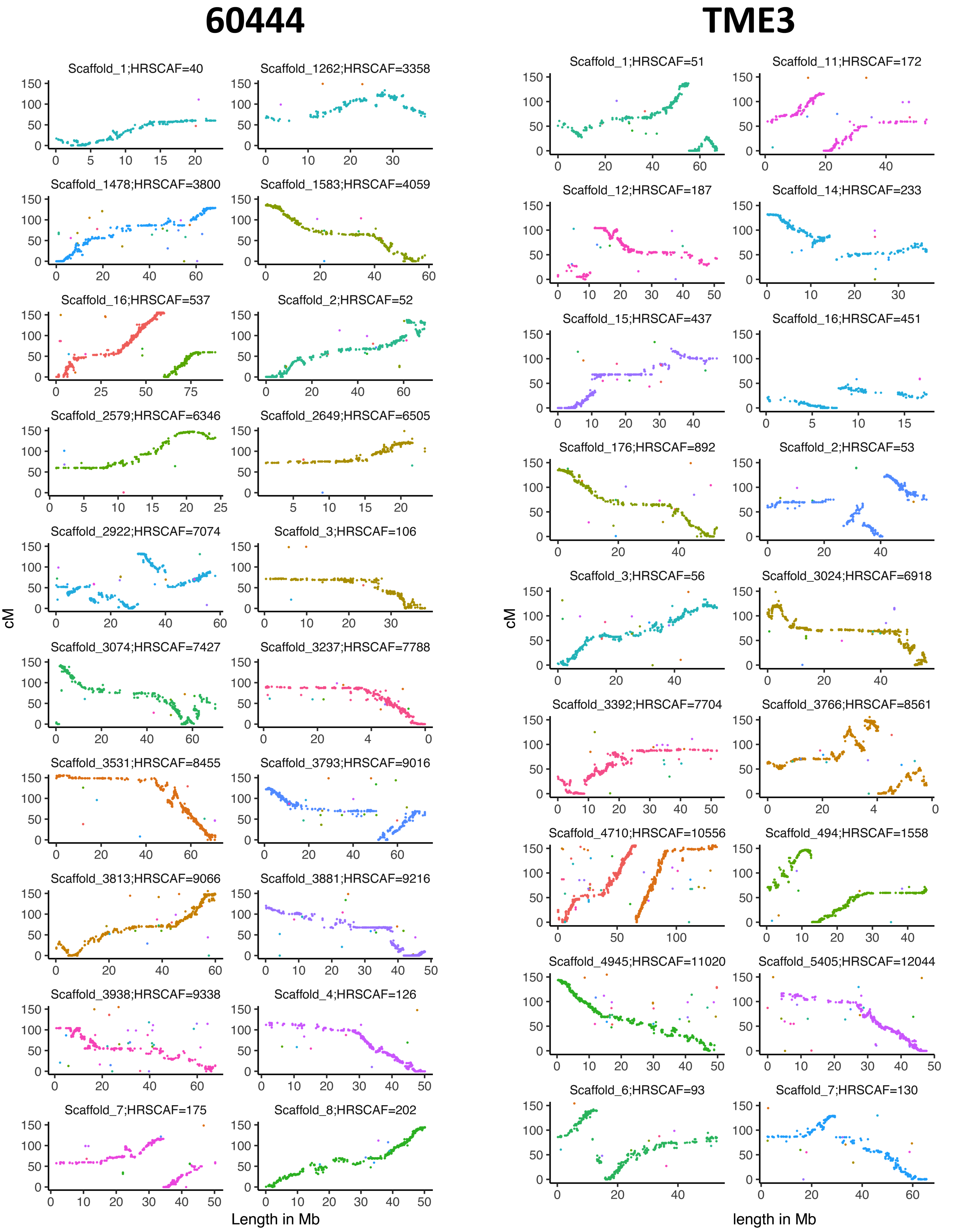
**

**Figure S4**

Pseudo-molecule validation using the 22,403 genetic markers from the cassava composite genetic map and the 18 pseudo-chromosomes of the cassava composite genetic map. Marker were aligned to each genome using BLAT. Each dot indicates a full-length sequence match. The x-axis represents the physical map of a HiRise scaffold and the y-axis the genetic distance extracted from the cassava composite genetic map [38]. Chromosomes were visualized with different colors. For chromosome identifiers see Table S12.

**
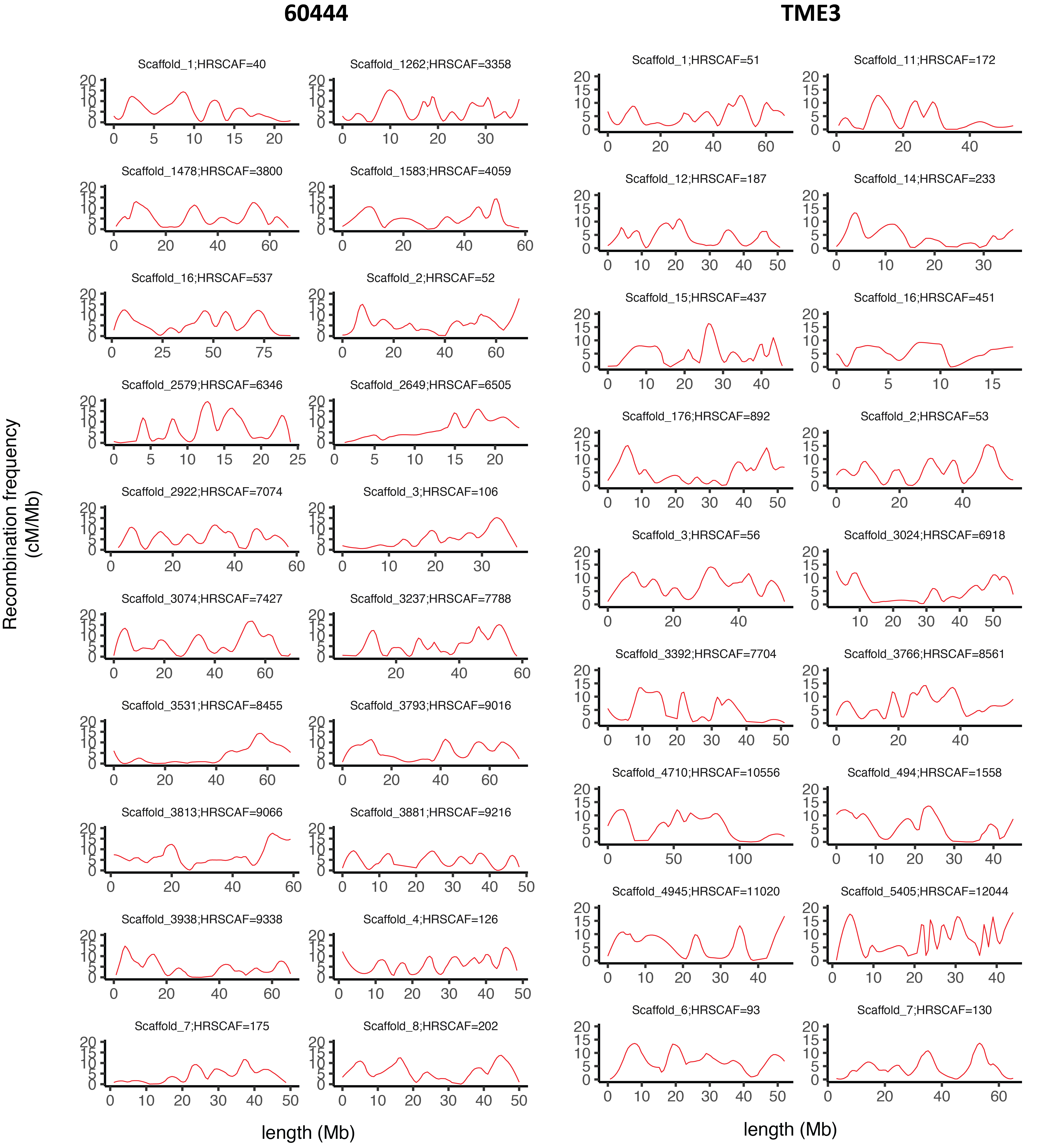
**

**Figure S5**

Recombination rates (in recombination per Mb) are plotted for the cassava chromosomal pseudo-molecules. For chromosome identifiers see Table S12.

**
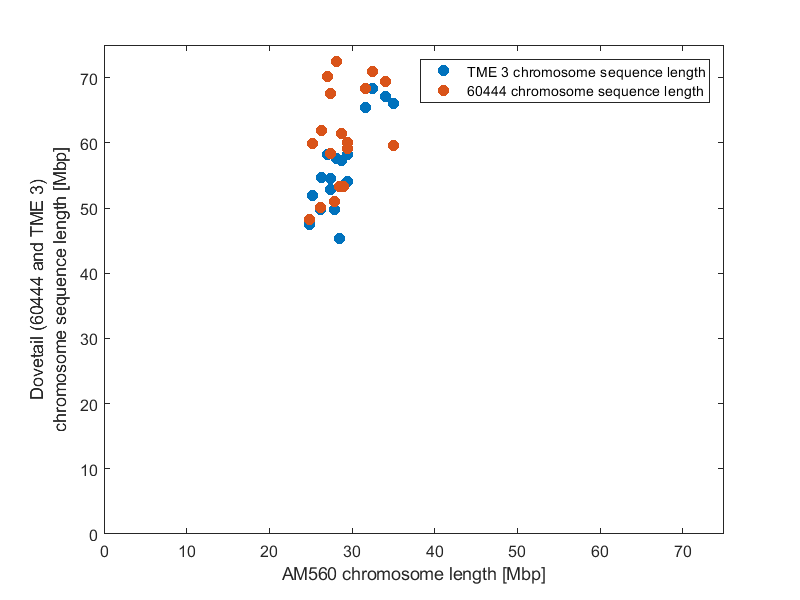
**

**Figure S6**

Plot of the length (Mbp) of the 18 chromosomes of AM560 compared to the combined length of the sequences that can be associated with the respective chromosomes in 60444 (red) and TME3 (blue).

**Figure S7**

Occurrence of genetic markers identified on TME3 (A, C, E) and 60444 (B, D, F) for CANU (A - B), CANU-BNG (C - D) and dovetail genomes (E - F) using blast with a cut off of 100% identity and 100% coverage. Occurrence is split into five classes: not found (white) found once (red) found more than one time on the same scaffold (blue) found more than one time on different scaffolds (green), found more than one time both on the same and on different scaffolds (black). Marker occurrences for more than 5 times exist, but represent rare cases.

**
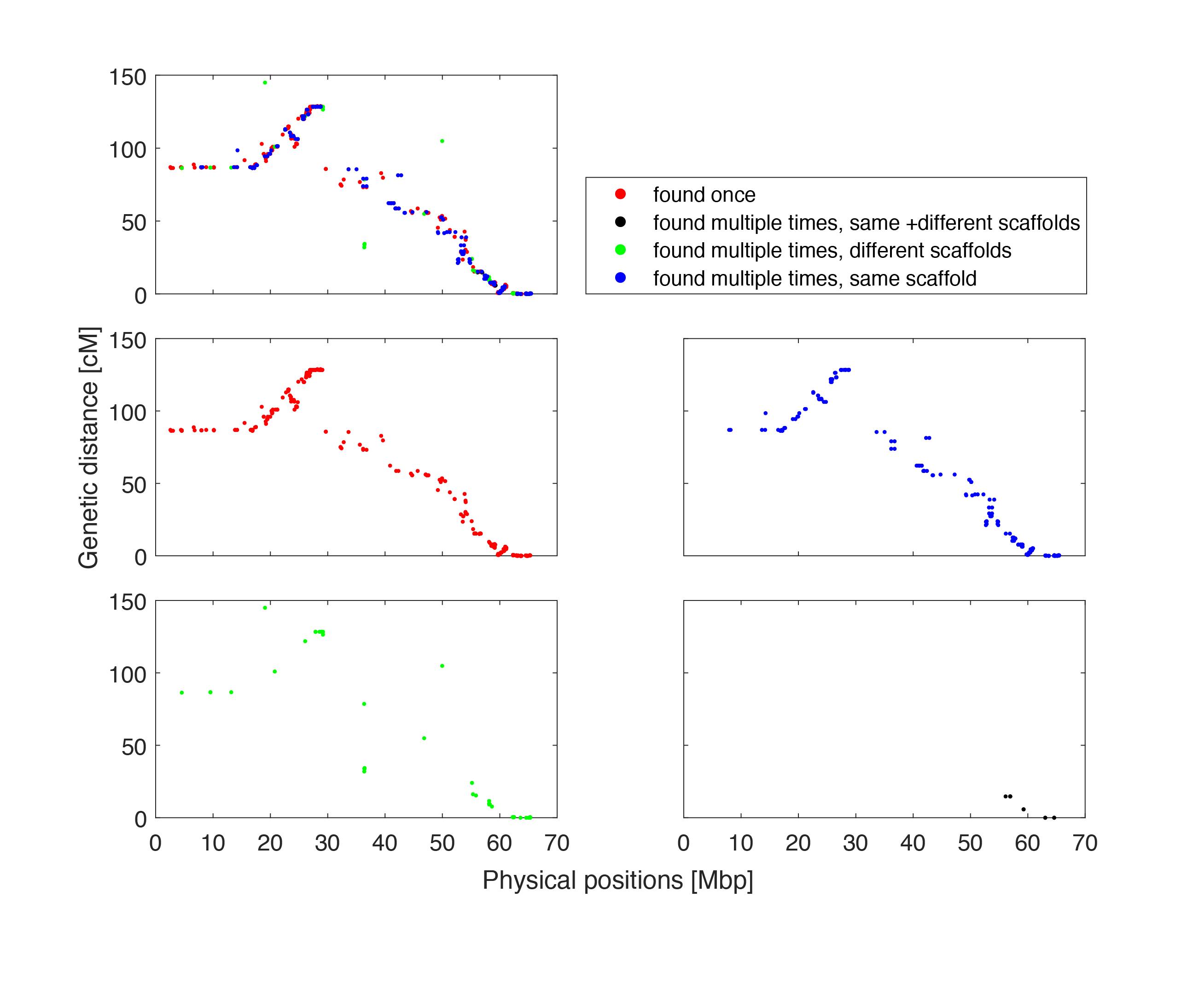
**

**Figure S8**

Genetic distance to physical distance plot of TME3 Scaffold 7, representing Chromosome 12 in AM560, is shown as an example. Genetic markers found only a single time in the chromosome (red), multiple times on the same scaffold (blue), multiple times on different scaffolds (green), and multiple times both on the same and different scaffolds (black) with individual plots for the different classifications.

**Figure S9**

Example of a mis-assembly identification using chromosome conformation capture read pairs. The paired-end mapping positions in the region 2,200,000-2,340,00 bp of Super-Scaffold_123 show a sudden absence of read pairs spanning across the region at around 2,280,000 bp. MQ: read mapping quality

**Figure S10**

Summary of full-length transcriptome sequencing for high-quality gene-space annotation. a) Length distribution and data density of full-length sequenced transcripts from 60444 and TME3 RNA. b) AED analysis of the gene model prediction. Plot shows the cumulative fraction of the annotations on the y-axis and the AED scores calculated by the annotation pipeline on the x-axis. Red line represents the updated annotation that used the Iso-Seq data and green line shows the AED scored for the genes annotated without Iso-Seq. c) Improved full-length transcript supported gene space annotation for the 60444 genome assembly. The top track shows the previous gene space annotation29 (Reference gene models Mesc.v6.1 annotations). The two tracks below (Polished Isoform reads) represent sequence alignments of full-length transcript reads of 60444 RNA. Blue and green arrow indicate the two sequenced alleles aligning to that locus. Black dots in Allele B represent indels and mutations, whereas Allele A aligns with no mismatch.

**Figure S11**

GO enrichment analysis for the genes specific to the AM560 genome

**Figure S12**

GO enrichment analysis for the genes specific to the 60444 and TME3 genome

**Figure S13**

Squalene monooxygenase activity (GO:0005198) pathway and the corresponding gene models found in 60444, TME3 and AM560


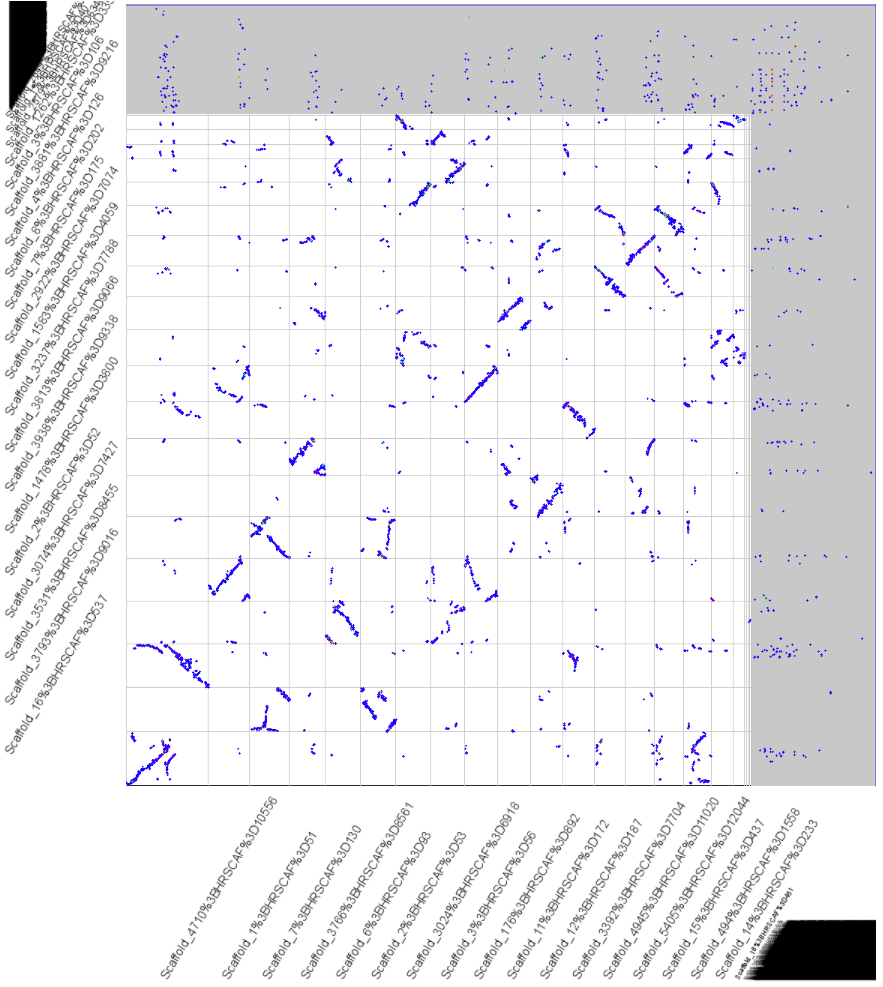


**Figure S14**

Syntenic dotplot generated by SynMap (<https://genomevolution.org/coge/>) between cassava 60444 and TME3. Each syntenic gene is depicted as dot. TME3 on the x-axis and 60444 is shown on the y-axis.

**Figure S15**

Syntenic relation of the long arm of chromosome 12 between the AM560 v6.1 genome and equivalent scaffolds of the TME3 or 60444 genomes. Light bars indicate syntenic gene alignments between the two sequences. Red lines indicate breaks in sequence contiguity. Cyan lines indicate genes that were *de novo* anchored to the TME3 and 60444 gene map.


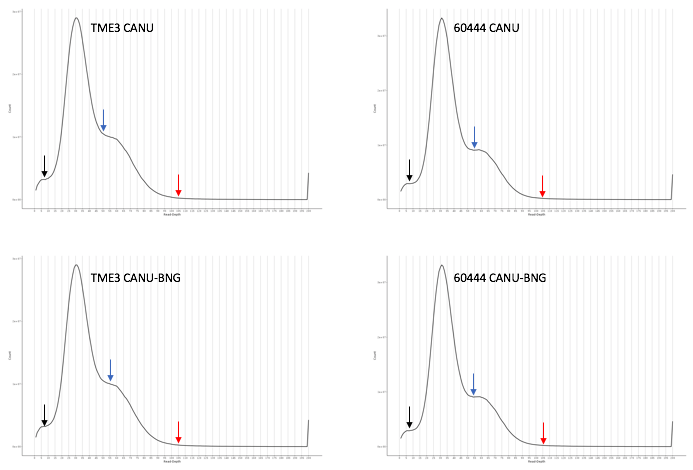


**Figure S16**

Read coverage histograms of TME3 and 60444 assemblies. The highest peak indicates haploid level of coverage, the second highest peak represents diploid level of coverage. The black, blue and red arrows mark the selected cutoff values for low, middle, and high coverage, respectively.
