# Supplementary material for: Haplotype-resolved genomes of geminivirus-resistant and geminivirus-susceptible African cassava cultivars
Source: BMC Biol. 2019 Sep 18;17:75. doi: 10.1186/s12915-019-0697-6 (PMC6749633; doi:10.1186/s12915-019-0697-6)
Supplement: Supplementary file 2 — Table S1. Assembly statistics of representative genome drafts from the three different assemblers. Table S2. Assembly accuracy evaluation using publicly available Illumina paired-end reads. Table S3. Optical map assembly using the IrysView software provided by BioNano and using option ‘optArguments_human’. Table S4. Structural variations based on optical maps of two cassava lines. Table S8. PacBio Iso-seq full length-transcriptome sequence classification. Table S9. Structural annotation of transposable elements in 60444 and TME3. Table S10. Non-coding RNA detected in the two cassava genomes. Table S11. BUSCO analysis of genome assemblies for 60444 and TME3. Table S12. Scaffolds representing the 18 pseudochromosomes of the cassava de novo genomes. Table S13. Hi-C library sequencing and read quality. Table S14. Hi-C read pair evaluation using HiCUP analysis pipeline (v0.5.8). (DOC 189 kb) [file 12915_2019_697_MOESM2_ESM.doc]

**Supplementary Tables**

**Table S1**

Assembly statistics of representative genome drafts from the three different assemblers.

|  |  | 60444 |  |  | TME3 |  |
| --- | --- | --- | --- | --- | --- | --- |
|  | Contigs | length (Mb) | N50 (Kb) | Contigs | length (Mb) | N50 (Kb) |
| CANU | 11,459 | 975 | 117 | 12,971 | 947 | 98 |
| FALCON | 10,428 | 1.058 | 134 | 12,280 | 992 | 119 |
| PBcR-MHAP | 22,547 | 812 | 45 | 33,277 | 854 | 32 |

**Table S2**

Assembly accuracy evaluation using publicly available Illumina paired-end reads

|  |  | Mapped  (%) | Both mapped (%) | Properly paired (%) | Discordant reads (%) | Total reads (in Mio.) |
| --- | --- | --- | --- | --- | --- | --- |
| 60444 | Falcon | 96 | 95 | 90 | 5.0 | 409 |
|  | Canu | 98 | 98 | 97 | 1.2 | 409 |
|  | PBcR-MHAP | 92 | 90 | 85 | 5.6 | 409 |
| TME3 | Falcon | 94 | 92 | 87 | 4.7 | 568 |
|  | Canu | 96 | 95 | 93 | 1.6 | 568 |
|  | PBcR-MHAP | 90 | 87 | 82 | 5.2 | 568 |

**Table S3**

Optical map assembly using the IrysView software provided by BioNano and using option 'optArguments_human'.

|  | TME3 | 60444 |
| --- | --- | --- |
| Mapped Molecule Quantity (Mb) | 64,060.011 | 70,148.008 |
| Mapped Avg Size (Kb) | 265 | 268 |
| Avg Label Density (per 100 Kb) | 9.7 | 9.5 |
| Number of Consensus Genome Maps | 952 | 926 |
| Consensus Genome Maps Size (Mb) | 1204.598 | 1204.106 |
| Haploid-genome size estimation (Mb) based on flow-cytometry | 765 | 745 |
| Consensus Genome Maps N50 (Mb) | 1.801 | 1.875 |

**Table S4**

Structural variations based on optical maps of two cassava lines

|  | | 60444 optical map (reference) vs TME3 optical map (query) |
| --- | --- | --- |
| Total size of genome map (Mb) TME3 | 1204.6 | |
| Map aligned to 60444 genome (Mb) | 974.3 | |
| Map uniquely aligned to 60444 genome (Mb) | 612.03 | |
| Region in TME3 with insertion and deletion (Mb) | 107.24 | |
| Ratio of region with insertion or deletion (%) | 8.9 | |
| Number of insertions | 1,058 | |
| Average insertion size (bp) | 57336.84 | |
| Number of deletions | 1,021 | |
| Average deletion size (bp) | 45615.34 | |

**Table S8**

PacBio Iso-seq full length-transcriptome sequence classification

|  | 60444 | TME3 |
| --- | --- | --- |
| Number of reads of insert | 181,785 | 296,047 |
| Number of five prime reads | 128,972 | 182,131 |
| Number of three prime reads | 133,388 | 187,096 |
| Number of poly-A reads | 123,772 | 173,033 |
| Number of filtered short reads | 6,028 | 16,526 |
| Number of non-full-length reads | 72,153 | 138,907 |
| Number of full-length reads | 103,604 | 140,614 |
| Number of full-length non-chimeric reads | 82,197 | 113,182 |
| Average full-length non-chimeric read length (bp) | 2,151 | 2,003 |
| Number of unpolished consensus transcripts | 52,810 | 71,004 |
| Average length of unpolished consensus transcripts (bp) | 2,256 | 2,116 |
| Number of polished high-quality (>99% accuracy) transcripts | 8,483 | 10,780 |
| Number of polished low-quality (<99% accuracy) transcripts | 44,274 | 60,082 |

**Table S9**

Structural annotation of transposable elements in 60444 and TME3

**Table S10**

Non-coding RNA detected in the two cassava genomes

|  |  | **60444** | **TME3** |
| --- | --- | --- | --- |
| **Non-coding RNA** | **Type** | **Copies** | **Copies** |
|  | rRNAs | 706 | 555 |
|  | tRNAs | 1,658 | 1,533 |
|  | miRNAs | 325 | 333 |
|  | snRNAs | 36 | 33 |

**Table S11**

BUSCO analysis of genome assemblies for 60444 and TME3

|  | 60444 | TME3 |
| --- | --- | --- |
| Complete BUSCOs | 1,369 (95%) | 1,364 (94.8%) |
| Complete and single-copy BUSCOs | 1,043 (72.4%) | 1,081 (75.4%) |
| Complete and duplicated BUSCOs | 326 (22.6%) | 283 (19.7%) |
| Fragmented BUSCOs | 20 (1.4%) | 19 (1.3%) |
| Missing BUSCOs | 51 (3.6%) | 57 (3.9%) |
| Total BUSCO groups searched | 1,440 | 1,440 |

**Table S12**

**Scaffolds representing the 18 pseudochromosomes of the cassava *de novo* genomes**

| **60444** | **Chr.** | **TME3** | **Chr.** |
| --- | --- | --- | --- |
| Scaffold_16;HRSCAF=537 | 1 | Scaffold_4710;HRSCAF=10556 | 1 |
| Scaffold_3531;HRSCAF=8455 | 2 | Scaffold_4710;HRSCAF=10556 | 2 |
| Scaffold_3813;HRSCAF=9066 | 3 | Scaffold_3766;HRSCAF=8561 | 3 |
| Scaffold_3;HRSCAF=106 | 4 | Scaffold_3024;HRSCAF=6918 | 4 |
| Scaffold_2649;HRSCAF=6505 | 4 | Scaffold_494;HRSCAF=1558 | 5 |
| Scaffold_2579;HRSCAF=6346 | 5 | Scaffold_4945;HRSCAF=11020 | 6 |
| Scaffold_16;HRSCAF=537 | 5 | Scaffold_6;HRSCAF=93 | 7 |
| Scaffold_8;HRSCAF=202 | 6 | Scaffold_1;HRSCAF=51 | 8 |
| Scaffold_3074;HRSCAF=7427 | 7 | Scaffold_176;HRSCAF=892 | 9 |
| Scaffold_2;HRSCAF=52 | 8 | Scaffold_3;HRSCAF=56 | 10 |
| Scaffold_1583;HRSCAF=4059 | 9 | Scaffold_14;HRSCAF=233 | 11 |
| Scaffold_1262;HRSCAF=3358 | 10 | Scaffold_16;HRSCAF=451 | 11 |
| Scaffold_1;HRSCAF=40 | 10 | Scaffold_7;HRSCAF=130 | 12 |
| Scaffold_2922;HRSCAF=7074 | 11 | Scaffold_2;HRSCAF=53 | 13 |
| Scaffold_1478;HRSCAF=3800 | 12 | Scaffold_15;HRSCAF=437 | 14 |
| Scaffold_3793;HRSCAF=9016 | 13 | Scaffold_5401;HRSCAF=12026 | 15 |
| Scaffold_3881;HRSCAF=9216 | 14 | Scaffold_11;HRSCAF=172 | 16 |
| Scaffold_4;HRSCAF=126 | 15 | Scaffold_12;HRSCAF=187 | 17 |
| Scaffold_7;HRSCAF=175 | 16 | Scaffold_3392;HRSCAF=7704 | 18 |
| Scaffold_3938;HRSCAF=9338 | 17 |  |  |
| Scaffold_3237;HRSCAF=7788 | 18 |  |  |

**Table S13**

**Hi-C library sequencing and read quality**

|  | 60444 | | TME3 | |
| --- | --- | --- | --- | --- |
|  | read 1 | read 2 | read 1 | read 2 |
| Total Reads | 385,205,561 | 385,205,561 | 390,910,077 | 390,910,077 |
| Not Truncated | 200,583,838 | 205,227,567 | 236,645,416 | 241,072,563 |
| Truncated | 184,621,723 | 179,977,994 | 154,264,661 | 149,837,514 |
| Unique Alignments | 74,810,656 | 73,698,941 | 81,460,833 | 80,178,437 |
| Multiple Alignments | 278,700,053 | 275,025,552 | 274,742,203 | 270,168,063 |
| Failed To Align | 20,394,334 | 25,429,065 | 25,425,706 | 31,532,642 |
| Paired | 27,241,726 | 27,241,726 | 32,609,953 | 32,609,953 |

**Table S14**

**Hi-C read pair evaluation using HiCUP analysis pipeline (v0.5.8)**

|  | HiCUP filtering | |
| --- | --- | --- |
|  | 60444 | TME 3 |
| Valid Pairs | 18,355,991 | 20,232,624 |
| Invalid Pairs | 8,885,735 | 12,377,329 |
| Same Circularised | 830,429 | 687,076 |
| Same Fragment Dangling Ends | 656,743 | 1,314,628 |
| Same Fragment Internal | 3,385,343 | 5,803,749 |
| Re-ligation | 952,106 | 1,476,814 |
| Contiguous Sequence | 80,407 | 122,003 |
| Wrong Size | 2,980,707 | 2,973,059 |
| Total Pairs | 27,241,726 | 32,609,953 |
