# Supplementary material for: Haplotype-resolved genomes of geminivirus-resistant and geminivirus-susceptible African cassava cultivars
Source: BMC Biol. 2019 Sep 18;17:75. doi: 10.1186/s12915-019-0697-6 (PMC6749633; doi:10.1186/s12915-019-0697-6)
Supplement: Supplementary file 3 — Supplementary Materials and Methods. (DOC 177 kb) [file 12915_2019_697_MOESM3_ESM.doc]

**Supplementary Materials and Methods**

**Plant material**

We sequenced *Manihot Esculenta* (cassava) cultivar TME3 (also known as Tropical Manihot Esculenta) and cultivar 60444. TME3 was originally collected in farmers’ fields of Nigeria and other West African countries during the 1980s and 1990s [1]. TME3 provides broad protection against all known cassava mosaic begomoviruses that cause cassava mosaic disease (CMD). Because dominant resistance can be easily tracked, *CMD2* became the predominant CMD resistance source in African cassava breeding programs, although its underlying molecular mechanism remains unknown [2,3]. Cassava cultivar 60444 is CMD-susceptible but is often considered a model cultivar because it can be efficiently transformed [4,5]. Genotyped shoot cultures of 60444 and TME3 were obtained from the ETH Zurich *in vitro* cassava germplasm collection.

**PacBio library preparation, PacBio RSII sequencing and PacBio assembly**

High-molecular weight (HMW) genomic DNA was extracted from three week-old plantlets grown on CBM medium in sterile conditions [4] using a modified CTAB method [6]. DNA integrity was assessed by standard agarose gel electrophoresis and Thermo Fisher Scientific Qubit Fluorometry (Invitrogen). PacBio 20kb SMRTbell libraries were prepared as recommended [7]. SMRT-Libraries were sequenced using a PacBio RSII long read sequencing instrument and P6C4 sequencing reagents. In total, we used 47 SMRT cells for 60444 and 45 SMRT cells for TME3. We generated 5,777,131 subreads for 60444 with a read length N50 of 12,813 Kbp and 52,4 Gbp total length. For TME3, we generated 7,650,003 subreads with 12,424 Kbp read length N50 and total length of 53,9 Gbp.

*De novo* assembly of the subreads was performed using three assemblers: the PBcR-MHAP (PBcR) pipeline (wgs-8.3rc2) [8], the CANU-MHAP (CANU-1.4) assembler [9] and the FALCON (v0.5) assemblers [10]. PBcR assembly was performed with the genome size set to 500 Mb for both genomes, estimated by the assembled size of the AM560 v6.1 reference genome. With the FALCON assembler, we performed parameter sweep and chose parameters to maximize contig N50. For CANU assembly, the estimated genome size was set to 527 Mb and 633 Mb for 60444 and TME3, respectively. Both values were estimated using k-mer analysis of Illumina paired-end reads (KAT-Release-2.1.1)[11]. Assembled drafts were benchmarked using Illumina paired-end data and reference gene models. Selected drafts were then polished using PacBio raw reads (in h5 format) with two rounds of quiver (SMRT Analysis 2.3.0) correction

**Genome assembly validation**

To assess the quality of the genome assemblies, publicly available paired-end (PE) short reads (60444 WGS:SRX1393211, TME3 WGS:SRX526747) were aligned to the respective drafts. In brief, sequencing adapters were trimmed using Trimmomatic (v.033) [12] and PE-reads were mapped using Burrows Wheel Alignment Tool bwa aln (v0.7.12) [13]. Mapping statistics were collected using Samtools (v1.3) [14] and Qualimap2 (v2.2.1) [15]. Of the 409,126,944 Illumina short reads from 60444, 98.3% were successfully mapped to the CANU assembly, with 96.6% properly paired. For TME3, we were able to map 96.4% of the 568,006,046 reads to the CANU assembly with 93,4% properly paired. For the FALCON assemblies, we received overall lower mapping values. For 60444, we mapped 96,1% with 90% properly paired reads. For TME3, we aligned 93.8% with 86% properly paired. The drafts produced by PBcR were very fragmented and had lowest Illumina read mapping rates. Thus, PBcR drafts were excluded from further analysis. We then generated an *in silico* restriction map for contigs from both CANU and FALCON assemblers according to the recognition signature of the nicking enzyme Nt.BspQI. The maps were aligned with corresponding optical maps generated from BioNano molecular data using RefAligner software (BioNano Genomics) to identify and curate potential conflicts in the contigs or in the optical maps. The results showed that the CANU assemblies had the lowest number of conflicts. When compared with FALCON assemblies, CANU introduced less assembly errors but produced a slightly more fragmented draft (lower N50 values). The parameter sweep aiming at largest N50 during FALCON assembly might have been too aggressive and thus increased error rate.

**Optical map construction**

Cassava plants were grown *in vitro* for three weeks and then placed in the dark for two days. HMW DNA was isolated according to the standard BioNano protocol ‘IrysPrep Plant Tissue DNA Isolation User Guide’. Briefly, DNA was digested using the single-stranded nicking endonuclease Nt.BspQI and labelled with a fluorescent dUTP nucleotide using *Taq* polymerase. The nicks were ligated using *Taq* DNA ligase and the DNA backbone stained with YOYO-1 dye (Thermo Fischer) for backbone staining. DNA imaging was done automatically using the BioNano Irys instrument. Molecules > 150 kb (and more than eight labels) were assembled into consensus optical maps using the BioNano IrysView analysis software. We used the IrysView pre-adjusted option ‘optArguments_human’ for assembly.

**Genome diversity analysis**

To compare the two assemblies on a genome-wide scale, we used the optical maps of the two cassava lines to detect structural variations (SVs) using the runBNG software [16] and the reference maps from 60444 and TME3 as queries. runBNG acts as a wrapper and essentially uses the BioNano program RefAligner for generating the alignments. Alignments were generated using the option ‘-z 1200Mb -t 1 -m 4’ screened by the script ‘SVDetect’ to detect the intergenomic SVs and to calculate insertion and deletion sizes [7]. Genome synteny between the two cassava genomes was analysed using the SynMap tools (CoGe, [www.genomevolution.org](http://www.genomevolution.org/)). To identify collinearity blocks using homologous CDS pairs the following parameters were applied: maximum distance between two matches (-D) was set to ‘20’. Minimum number of aligned pairs (-A) was set to ‘10’. The algorithm ‘Quota Align Merge’ (github.com) was set with maximum distance between two blocks (-Dm) ‘500’.

**Three-dimensional genome-wide chromatin capture sequencing**

We used five grams of fresh leaves from *in vitro*-grown TME3 and 60444 plantlets, which had been placed in the dark for 48h before harvest. The leaf material was vacuum-infiltrated in nuclei isolation buffer (NIB) supplemented with 2% formaldehyde. Protein-crosslinking was stopped by adding glycine and applying an additional vacuum infiltration step. Leaf tissue was snap-frozen using liquid nitrogen and ground into a fine powder, re-suspend in NIB and purified by spin-downs as described earlier [17]. Nuclei were digested with 400 units of *HindIII* [17]. Digested chromatin was labelled using a fill-in reaction with 60 units of Klenow polymerase and biotin-14-dCTP. The exonuclease activity of T4 DNA polymerase was used to remove biotin-14-dCTP from non-ligated DNA ends. Proteinase K was added to reverse the formaldehyde cross-linking and DNA was purified following phenol-chloroform extraction as described earlier [17]. The Hi-C samples were quality assessed by PCR amplification of a 3C template and evaluated as published [17] (Additional File 1: Figure S3). Quality control passed Hi-C samples were purified using a phenol-chloroform extraction protocol [17] and mechanically sheared to fragment sizes of 300 bp using the Covaris S2 ultrasonicator. Hi-C library fragments were blunt-ended using the End Repair Mix from Illumina and purified using the AMPure beads according to the standard AMPure protocol (Beckman Coulter). The biotinylated Hi-C samples were enriched through biotin-streptavidin-mediated pull-down and adenylated using Illumina’s A-tailing mix. Illumina paired-end sequencing adaptors were ligated to the Hi-C fragments and a PCR amplification of the Hi-C library was carried out according to the Illumina protocol. Finally, PCR products were purified using AMPure beads and the standard AMPure protocol and quantified using a Q-bit device. Samples were sequenced using the Illumina HiSeq 4000 instrument. This produced 385 million 151 bp paired-end reads for 60444 and 391 million reads for TME3 providing 51.3x and 52.1x physical coverage, respectively. To assess the quality of the Hi-C sequencing, sequence reads were quality filtered using the HiCUP pipeline (v0.5.8), a software specifically designed to filter proper Hi-C read pairs from paired-end read contaminations [18]. This revealed 17.9 million unique and valid Hi-C pairs for 60444 and 20 million valid pairs for TME3.

**Scaffolding the PacBio and BioNano assemblies with HiRise**

Hi-C sequence data was used to scaffold the two cassava assemblies using HiRise, a software pipeline designed for using proximity ligation data to assemble sequences into chromosomal pseudo-molecules [19]. The mapping locations of Hi-C read pairs were analyzed by Dovetail using their proprietary HiRise pipeline to cluster sequences into large proximity bins. The read-pair position was also used to identify putative assembly errors.

**Genome size and heterozygosity estimation**

We measured the nuclear DNA content of the two cassava genotypes by flow cytometry. Two week-old *in vitro*-grown plants were processed together with the reference standard tomato (Lycopersium esculentum, cv. Stupice with genome size of 958 Mb) [20] and an internally used *Arabidopsis halleri* standard (not shown). The cassava haploid genome size was estimated according to a relative peak position using the propidium iodine-based CyStain PI absolute P kit and CyFlow Space multi-laser flow cytometer system (Sysmex Partec). We estimated a haploid genome size of 745 Mb for 60444 and 768 Mb for TME3 (Additional File 1: Figure S2). To assess the heterozygosity of the two cassava lines, we used the publicly available Illumina paired-end 100 bp sequencing reads from 60444, TME3 and the partly-inbred cassava reference genome AM560-2 ([https://phytozome.jgi.doe.gov/pz/portal.html#!info?alias=Org_Mesculenta](https://phytozome.jgi.doe.gov/pz/portal.html#!info?alias=Org_Mesculenta)), which were downloaded from NCBI Short Read Archive (SRX1393211, SRX526747, SRX1393218). Illumina reads were trimmed using the Trimmomatic tools [11]. Genome properties were analysed using SGA (v0.10.15) Preqc [21] with default parameters**.**

**Reassignment of allelic sequences**

Both 60444 and TME3 are heterozygous genomes and for regions of high heterozygosity two haplotypes exist within the assembled sequences. We identified the sequences that represent the two different haplotypes and assigned all sequences in the PacBio contigs as well as PacBio and BioNano hybrid assemblies using ‘Purge Haplotigs’ [22]. In brief, PacBio subreads were aligned back to the genome draft using minimap2 (v2.15) [23] and coverage histograms were generated using the ‘read_hist’ function. Read depth cut off values (low, middle and high) were determined by inspecting the histograms manually (Additional File 1: Figure S16). Coverage stats were generated using the function ‘contigcov’ and allelic sequences were assigned as primary and secondary haplotigs using the function ‘purge’.

**Iso-Seq RNA preparation and sequencing**

For full-length transcript sequencing, RNA was extracted from the following tissues of greenhouse-grown 60444 and TME3 plants: top five leaves with petioles, the apical meristem, lateral meristems, stems and roots. Tissues were snap-frozen in liquid nitrogen and ground using a mortar and pestle. RNA was isolated using a modified protocol [24] and RNA integrity was tested using an Agilent 2100 BioAnalyzer and Qubit Fluorometry (Invitrogen). A subset of the RNA samples was pooled and processed according to the PacBio Protocol: Procedure & Checklist – Iso-Seq Template Preparation for Sequel Systems (11/2017). The optimal number of cycles for large-scale PCR was determined to be 14. Amplification was followed by molecule size selection using 1x AMPure beads and 0.4x AMPure beads. The two purified fractions were pooled for library construction. We used one SMRT cell for each cassava line and sequenced using the PacBio Sequel instrument. A total of 181,823 reads covering 2,779,884,989 bp and 296,109 reads covering 3,768,451,277 bp were produced for 60444 and TME3 RNA libraries, respectively. The raw sequencing reads were processed using the Iso-seq protocol within SMRTlink (v.5.0.1.9585) to obtain full-length transcripts (Additional File 2: Table S8), which were error corrected using the Arrow algorithms provided by PacBio. Isoforms were aligned to the corresponding cassava genome using GMAP with option ‘-f samse’ and ‘-z sense_force’ and ‘-n 0’ [25]. The isoform alignments were used as input for the gene model annotation as described in the chapter ‘Gene Space Annotation’.

**DNA repeat sequence annotation and characterization**

DNA repeat families found in the two cassava genome assemblies were first independently identified *de novo* and classified using the software tool RepeatModeler [26]. RepeatModeler uses the programs RECON and the package RepeatScout for the *de novo* identification of repeats. After the classification process, the output data file from each of the genome assemblies was used as a custom repeat library by RepeatMasker [27] for the discovery and annotation of repetitive DNA elements. Detailed results are shown in Additional File 2: Table S9.

**Gene space annotation**

Protein coding genes were annotated using iterative MAKER (version 2.31.8) analysis. In the initial analysis, Augustus (version 3.2.3) [28] was trained with the cassava reference gene models and used for the *ab initio* prediction of gene models. Predictions were also based on evidence from three different alignments, including publically available cassava ESTs from NCBI, the full-length transcripts generated from Iso-Seq and UniProt protein sequences [29], to produce the initial set of gene models. These models from the first MAKER prediction were used to train the *ab initio* gene predictor SNAP (version 0.15.4, omicX), which was added in the second round of MAKER analysis. At this step, the *ab initio* gene predictor GeneMark (version 3.47) (<http://exon.gatech.edu/GeneMark/>) trained with Iso-seq data was also included. The final gene models were annotated using evidence from six different sources: the gene models from Augustus, SNAP and GeneMark, the cassava ESTs, the full-length transcriptome sequences and UniProt protein sequences. To assess the quality of the gene prediction, the AES scores were generated for each of the predicted genes throughout the annotation pipeline. Genes were further characterized for their putative function by performing BLASTp [30] searches against the UniProt database. Gene Ontology (GO) annotation was performed using InterProScan (version 5.27-66.0) (<https://www.ebi.ac.uk/interpro/search/sequence-search>). To annotate non-protein coding genes, the tools tRNASCAN-SE [31] (Version 2.0) and INFERNAL [32] (Version 1.1.2) were used with the Rfam database (version 13.0; [http://rfam.xfam.org](http://rfam.xfam.org/)) (Additional File 2: Table S10). Genome assembly and annotation completeness was assessed using the embryophyta_odb9 database of 1,440 single copy orthologs (<https://busco.ezlab.org/frame_wget.html>) using BUSCO [33] run with option ‘-m genome -long’ (Additional File 2: Table S11).

**OrthoMCL clustering and GO enrichments or under-representation**

Gene clusters were generated from the annotated gene set of the three cassava genomes 60444, TME3 and AM560, *Ricinus communis* and *Arabidopsis* using the OrghoMCL software tools (v2.0) [34] (<https://phytozome.jgi.doe.gov/pz/portal.html>). Splice variants were removed from the protein data set and proteins were filtered for internal stop codons. The input dataset comprised 33,853 TME3 proteins, 34,127 60444 proteins and 33,033 AM560 proteins. First, pairwise sequence similarities between all input coding sequences were defined using BLASTP and a e-value cut-off of 1e-05. Markov clustering was used to define the ortholog cluster structure using the default inflation value of 1.5. A total of 101,013 proteins from the three different genomes were clustered into 17,648 gene families. A set of 11,910 clusters contained coding sequences from all three cassava genomes.

Cultivar-specific genes and genes shared between 60444 and TME3 were extracted from clusters and tested for GO enrichments or under-representations using a hypergeometric testing available in the GOstats and GSEABase function from the Bioconductor R package [35]. The REVIGO tool [36] was used to remove redundant terms from long GO lists and to visualize enrichment results.

**Allele-specific expression analysis**

For deep transcriptome sequencing, cassava 60444 plant organs were collected at three key developmental stages as follows: the top-3 leaves and fibrous roots (FR) from 4 week-old plants, the top-3 leaves, FR and early storage roots (IR) from 8 week-old plants, and the top-3 leaves, FR, IR and growing storage root (SR) from 12 week-old plants, with three independent replicates per organ. RNA samples were prepared according to a modified protocol [24] and tested for integrity using Qubit Fluorometry (Invitrogen) and the Bioanalyzer 2100 (Agilent). High-throughput sequencing was performed on an Illumina HiSeq 2000 instrument run in paired-end 2x100 nucleotides mode. Reads were processed with Trimmomatic (v.35) [12] to remove adapter and low quality sequences (< 20 bp quality). Reads were mapped to the 60444 genome assembly using STAR (v2.5.3a) [37] and read duplicates marked using the ‘-bamRemoveDuplicates’ with type ‘UniqueIdentical’. Unspecific reads were removed from the mapping file using SAMtools [14] with option ‘view -F0x400’. Allelic gene space was annotated using *de novo* CDSs aligned to the genome assembly using GMAP [25] run with the option ‘-nofails -min-identity=0.5 -f1’. Alignment positions were extracted using custom scripts and RNAseq reads counted using the SAMtools wrapper pysam and the module ‘fetch’ (<https://github.com/pysam-developers/pysam>) and custom python scripts. An expression ratio was calculated by dividing FPKM_alleleA by FPKM_alleleB. Mono-allelic expressed genes were defined when this expression ratio was < 0.25.

**References**

1. Okogbenin E, Egesi CN, Olasanmi B, Ogundapo O, Kahya S, Hurtado P, et al. Molecular marker analysis and validation of resistance to cassava mosaic disease in elite cassava genotypes in Nigeria. Crop Sci. 2012;52:2576–86.

2. Rabbi IY, Hamblin MT, Kumar PL, Gedil M a., Ikpan AS, Jannink JL, et al. High-resolution mapping of resistance to cassava mosaic geminiviruses in cassava using genotyping-by-sequencing and its implications for breeding. Virus Res. Elsevier; 2014;186:87–96.

3. Wolfe MD, Rabbi IY, Egesi C, Hamblin M, Kawuki R, Kulakow P, et al. Genome-wide association and prediction reveals the genetic architecture of cassava mosaic disease resistance and prospects for rapid genetic improvement. Plant Genome. 2016;9:1–13.

4. Bull SE, Owiti JA, Niklaus M, Beeching JR, Gruissem W, Vanderschuren H. Agrobacterium-mediated transformation of friable embryogenic calli and regeneration of transgenic cassava. Nat Protoc. Nature Publishing Group; 2009;4:1845–54.

5. Taylor NJ, Masona M V., Carcamo R, Ho T, Schöpke C, Fauquet CM. Production of embryogenic tissues and regeneration of transgenic plants in cassava (Manihot esculenta Crantz). Euphytica. 2001;120:25–34.

6. Doyle JJ, Doyle JL. A rapid total DNA preparation procedure for fresh plant tissue. Focus (Madison). 1990;12:13–5.

7. Pendleton M, Sebra R, Pang AWC, Ummat A, Franzen O, Rausch T, et al. Assembly and diploid architecture of an individual human genome via single-molecule technologies. Nat Methods. 2015;12:780–6.

8. Berlin K, Koren S, Chin C-S, Drake JP, Landolin JM, Phillippy AM. Assembling large genomes with single-molecule sequencing and locality-sensitive hashing. Nat Biotechnol. 2015;33.

9. Walenz BP, Koren S, Bergman NH, Phillippy AM, Miller JR, Berlin K. Canu: scalable and accurate long-read assembly via adaptive k -mer weighting and repeat separation. Genome Res. 2017;27:722–36.

10. Chin C, Peluso P, Sedlazeck FJ, Nattestad M, Concepcion GT, Clum A, et al. Phased diploid genome assembly with single-molecule real-time sequencing. Nat Methods. 2016;13.

11. Mapleson D, Garcia Accinelli G, Kettleborough G, Wright J, Clavijo BJ. KAT: a K-mer analysis toolkit to quality control NGS datasets and genome assemblies. Bioinformatics. 2016/11/28. Oxford University Press; 2017;33:574–6.

12. Bolger AM, Lohse M, Usadel B. Genome analysis Trimmomatic : a flexible trimmer for Illumina sequence data. Bioinformatics. 2014;30:2114–20.

13. Li H, Durbin R. Fast and accurate short read alignment with Burrows – Wheeler transform. Bioinformatics. 2009;25:1754–60.

14. Li H, Handsaker B, Wysoker A, Fennell T, Ruan J, Homer N, et al. The Sequence Alignment / Map format and SAMtools. Bioinformatics. 2009;25:2078–9.

15. Okonechnikov K, Conesa A, Garcı F. Genome analysis Qualimap 2 : advanced multi-sample quality control for high-throughput sequencing data. Bioinformatics. 2016;32:292–4.

16. Yuan Y, Bayer PE, Lee H, Edwards D. Sequence analysis runBNG : a software package for BioNano genomic analysis on the command line. Bioinformatics. 2017;1–3.

17. Grob S, Grossniklaus U. Chromatin Conformation Capture-Based Analysis of Nuclear Architecture. In: Kovalchuk I, editor. Plant Epigenetics Methods Protoc. Boston, MA: Springer US; 2017. p. 15–32.

18. Wingett S, Ewels P, Furlan-magaril M, Nagano T, Schoenfelder S, Fraser P, et al. HiCUP : pipeline for mapping and processing Hi-C data. F1000Research. 2015;1310:1–12.

19. Putnam NH, Connell BO, Stites JC, Rice BJ, Hartley PD, Sugnet CW, et al. Chromosome-scale shotgun assembly using an in vitro method for long-range linkage. Genome Res. 2016;26:1–25.

20. Dolezel J, Sgorbati S, Lucretti S. Comparisoii of three DNA fliiorocliromes for flow cytometric estimatioe of nuclear DNA conteet in plants. 1992;625–31.

21. Simpson JT, Durbin R. Efficient construction of an assembly string graph using the FM-index. Bioinformatics. 2010;26:i367–73.

22. Roach MJ, Schmidt SA, Borneman AR. Purge Haplotigs: allelic contig reassignment for third-gen diploid genome assemblies. BMC Bioinformatics. 2018;19:460.

23. Li H. Minimap2: pairwise alignment for nucleotide sequences. Bioinformatics. 2018;34:3094–100.

24. Chang S, Puryear J, Cairney J. A simple and efficient method for isolating RNA from pine trees. Plant Mol Biol Report. 1993;11:113–6.

25. Wu TD, Watanabe CK. GMAP: a genomic mapping and alignment program for mRNA and EST sequences. Bioinformatics. 2005;21:1859–75.

26. Smit AF., Hubley R. RepeatModeler Open-1.0. http://www.repeatmasker.org.

27. Smit AFA, Hubley R, Green P. RepeatMasker Open-4.0. http://www.repeatmasker.org.

28. Stanke M, Diekhans M, Baertsch R, Haussler D. Sequence analysis Using native and syntenically mapped cDNA alignments to improve de novo gene finding. Bioinformatics. 2008;24:637–44.

29. Apweiler R. UniProt: the Universal Protein knowledgebase. Nucleic Acids Res. 2004;32:115D – 119.

30. Altschul SF, Gish W, Miller W, Myers EW, Lipman DJ. Basic local alignment search tool. J Mol Biol. 1990;215:403–10.

31. Lowe TM, Chan PP. tRNAscan-SE On-line: integrating search and context for analysis of transfer RNA genes. Nucleic Acids Res. 2016;44:54–7.

32. Nawrocki EP, Eddy SR. Infernal 1.1: 100-fold faster RNA homology searches. Bioinformatics. 2013;29:2933–5.

33. Sima FA, Waterhouse RM, Ioannidis P, Kriventseva E V, Zdobnov EM. Genome analysis BUSCO : assessing genome assembly and annotation completeness with single-copy orthologs. Bioinformatics. 2015;31:3210–2.

34. Li L, Stoeckert CJJ, Roos DS. OrthoMCL: Identification of Ortholog Groups for Eukaryotic Genomes. Genome Res. 2003;13:2178–89.

35. Gentleman R, Carey V, Bates D, Bolstad B, Dettling M, Dudoit S, et al. Bioconductor: open software development for computational biology and bioinformatics. Genome Biol. 2004;5:R80.

36. Supek F, Bošnjak M, Škunca N, Šmuc T. Revigo summarizes and visualizes long lists of gene ontology terms. PLoS One. 2011;6.

37. Dobin A, Davis CA, Schlesinger F, Drenkow J, Zaleski C, Jha S, et al. STAR: Ultrafast universal RNA-seq aligner. Bioinformatics. 2013;29:15–21.

38. (ICGMC) ICGMC. High-Resolution Linkage Map and Chromosome-Scale Genome Assembly for Cassava (Manihot esculenta Crantz) from Ten Populations. G3. 2015;5:133–44.
